# Network-based analysis of prostate cancer cell lines reveals novel marker gene candidates associated with radioresistance and patient relapse

**DOI:** 10.1371/journal.pcbi.1007460

**Published:** 2019-11-04

**Authors:** Michael Seifert, Claudia Peitzsch, Ielizaveta Gorodetska, Caroline Börner, Barbara Klink, Anna Dubrovska

**Affiliations:** 1 Institute for Medical Informatics and Biometry (IMB), Carl Gustav Carus Faculty of Medicine, Technische Universität Dresden, Dresden, Germany; 2 National Center for Tumor Diseases (NCT), Partner Site Dresden, Germany; 3 OncoRay - National Center for Radiation Research in Oncology, Faculty of Medicine and University Hospital Carl Gustav Carus, Technische Universität Dresden, Helmholtz-Zentrum Dresden-Rossendorf (HZDR), Dresden, Germany; 4 Institute for Clinical Genetics, Carl Gustav Carus Faculty of Medicine, Technische Universität Dresden, Dresden, Germany; 5 Helmholtz-Zentrum Dresden-Rossendorf (HZDR), Institute of Radiooncology-OncoRay, Dresden, Germany; 6 German Cancer Consortium (DKTK) Partner Site Dresden, Germany, and German Cancer Research Center (DKFZ), Heidelberg, Germany; University of Virginia, UNITED STATES

## Abstract

Radiation therapy is an important and effective treatment option for prostate cancer, but high-risk patients are prone to relapse due to radioresistance of cancer cells. Molecular mechanisms that contribute to radioresistance are not fully understood. Novel computational strategies are needed to identify radioresistance driver genes from hundreds of gene copy number alterations. We developed a network-based approach based on lasso regression in combination with network propagation for the analysis of prostate cancer cell lines with acquired radioresistance to identify clinically relevant marker genes associated with radioresistance in prostate cancer patients. We analyzed established radioresistant cell lines of the prostate cancer cell lines DU145 and LNCaP and compared their gene copy number and expression profiles to their radiosensitive parental cells. We found that radioresistant DU145 showed much more gene copy number alterations than LNCaP and their gene expression profiles were highly cell line specific. We learned a genome-wide prostate cancer-specific gene regulatory network and quantified impacts of differentially expressed genes with directly underlying copy number alterations on known radioresistance marker genes. This revealed several potential driver candidates involved in the regulation of cancer-relevant processes. Importantly, we found that ten driver candidates from DU145 (*ADAMTS9*, *AKR1B10*, *CXXC5*, *FST*, *FOXL1*, *GRPR*, *ITGA2*, *SOX17*, *STARD4*, *VGF*) and four from LNCaP (*FHL5*, *LYPLAL1*, *PAK7*, *TDRD6*) were able to distinguish irradiated prostate cancer patients into early and late relapse groups. Moreover, in-depth *in vitro* validations for *VGF* (Neurosecretory protein VGF) showed that siRNA-mediated gene silencing increased the radiosensitivity of DU145 and LNCaP cells. Our computational approach enabled to predict novel radioresistance driver gene candidates. Additional preclinical and clinical studies are required to further validate the role of *VGF* and other candidate genes as potential biomarkers for the prediction of radiotherapy responses and as potential targets for radiosensitization of prostate cancer.

## Author summary

Prostate cancer cell lines represent an important model system to characterize molecular alterations that contribute to radioresistance, but irradiation can cause deletions and amplifications of DNA segments that affect hundreds of genes. This in combination with the small number of cell lines that are usually considered does not allow a straight-forward identification of driver genes by standard statistical methods. Therefore, we developed a network-based approach to analyze gene copy number and expression profiles of such cell lines enabling to identify potential driver genes associated with radioresistance of prostate cancer. We used lasso regression in combination with a significance test for lasso to learn a genome-wide prostate cancer-specific gene regulatory network. We used this network for network flow computations to determine impacts of gene copy number alterations on known radioresistance marker genes. Mapping to prostate cancer samples and additional filtering allowed us to identify 14 driver gene candidates that distinguished irradiated prostate cancer patients into early and late relapse groups. In-depth literature analysis and wet-lab validations suggest that our method can predict novel radioresistance driver genes. Additional preclinical and clinical studies are required to further validate these genes for the prediction of radiotherapy responses and as potential targets to radiosensitize prostate cancer.

## Introduction

Radiation therapy and surgery with or without anti-androgen treatment are key therapies for prostate carcinoma. Depending on the stage of tumor and type of applied irradiation, up to 90% of prostate cancer patients can be permanently cured by radiotherapy [1–3]. Nevertheless, normal tissue toxicity limits the delivery of a tumor curative radiation dose and is therefore one of the major obstacles to effective external beam radiotherapy [4]. Local recurrence of prostate cancer after radiotherapy can be attributed to radioresistance of cancer cells [5]. Molecular mechanisms and cellular properties that contribute to radioresistance of prostate cancer are only partly understood involving activations of signaling pathways such as PI3K/Akt and mTOR, alterations of DNA repair pathways, autophagy, and epithelial-mesenchymal transition, and the potential existence of cancer stem cells [5]. Another important factor involved in radioresistance of prostate cancer is the tumor microenvironment [6, 7]. Tumor progression and therapy response can be influenced by changes of the tumor microenvironment as a consequence of a radiation therapy [8, 9]. Closely related to this are immunomodulatory alterations triggered by radiation therapies that offer possibilities for new treatment options [10–12]. Also changes of the metabolism of cancer cells after a radiotherapy can alter the radiosensitivity of cells [13]. Still, the occurrence of radioresistance is highly unpredictable leading to less effective treatments for many patients supporting local recurrence and metastasis of prostate cancer [14]. Adjuvant therapies to further improve the efficiency of radiation therapies are urgently needed. Different molecular mechanisms and various agents have already been identified to improve the radiosensitization of prostate cancer. This includes androgen deprivation therapy, vascular endothelial growth factor (VEGF) inhibition, mTOR inhibition, and cytochrome P450 enzyme CYP17A1 inhibition [15]. Several other potential adjuvant strategies have also been suggested including the application of a Bcl-2 inhibitor [16], cytolethal distending toxin [17], PARP inhibition [18], resveratrol [19], and an ATM kinase inhibitor [20] to improve radiosensitization. However, additional molecular characterizations and studies are necessary to enable a targeted transfer into the clinics to further improve the efficiency of radiation therapies.

Still, clinical, pathological and biological factors for the prediction of treatment outcomes are of great importance for the personalization of prostate cancer treatment. The current pre-treatment risk stratification system for prostate cancer is based on the analysis of prostate-specific antigen, clinical T-stage and Gleason scores to guide medical decision making [21]. This concept for risk assessment of prostate cancer is of a high clinical value, but limited by the heterogeneity of patients within disease-risk groups [22]. Therefore, novel prognostic factors are required to obtain more accurate risk estimations for radioresistance.

Over the last years, different large-scale studies were performed to obtain a better general characterization of prostate cancer at the molecular level. This has contributed to the identification of molecular subtypes, recurrent gene mutations and DNA copy number alterations, and the characterization of signaling and DNA repair pathways involved in the development of prostate cancer (e.g. [23–26]). Especially the multi-omics study by The Cancer Genome Atlas (TCGA) [23] provides omics profiles of different molecular layers along with clinical information for hundreds of prostate cancer patients. Such data sets represent an important basis to gain novel insights into genes and molecular mechanisms driving radioresistance, but this search for novel candidate genes is very challenging comparable to the search for the needle in the haystack.

Irradiation of prostate cancer cells causes DNA double strand breaks and cells that survive this highly toxic damage can show complex genomic alterations such as large deletions or amplifications of chromosomal regions due to error-prone DNA repair [27]. Many genes are located in such altered regions and these altered regions differ substantially between radioresistant cells. Therefore, an identification of radioresistance drivers by standard statistical approaches is nearly impossible. Innovative computational concepts are required to separate potential drivers from passengers. A promising strategy is the analysis of gene dosage effects triggered by underlying deletions and amplifications with the help of gene regulatory networks [28–30]. This strategy is related to network-based stratification of gene mutations [31, 32]. We recently demonstrated that gene regulatory networks learned from gene expression and copy number profiles of cancer cell lines or cancer patients are capable to predict impacts of gene copy number alterations on cancer-relevant target genes, signaling pathways and patient survival [28–30]. The key principle behind this approach is the usage of a specifically designed network propagation algorithm to propagate gene expression alterations along the edges of a gene regulatory network to quantify how individual gene copy number alterations influence the expression of other genes. This concept can be adapted to the analysis of radioresistant prostate cancer cell lines offering the great opportunity to identify novel candidate genes involved in radioresistance.

**Fig 1:**
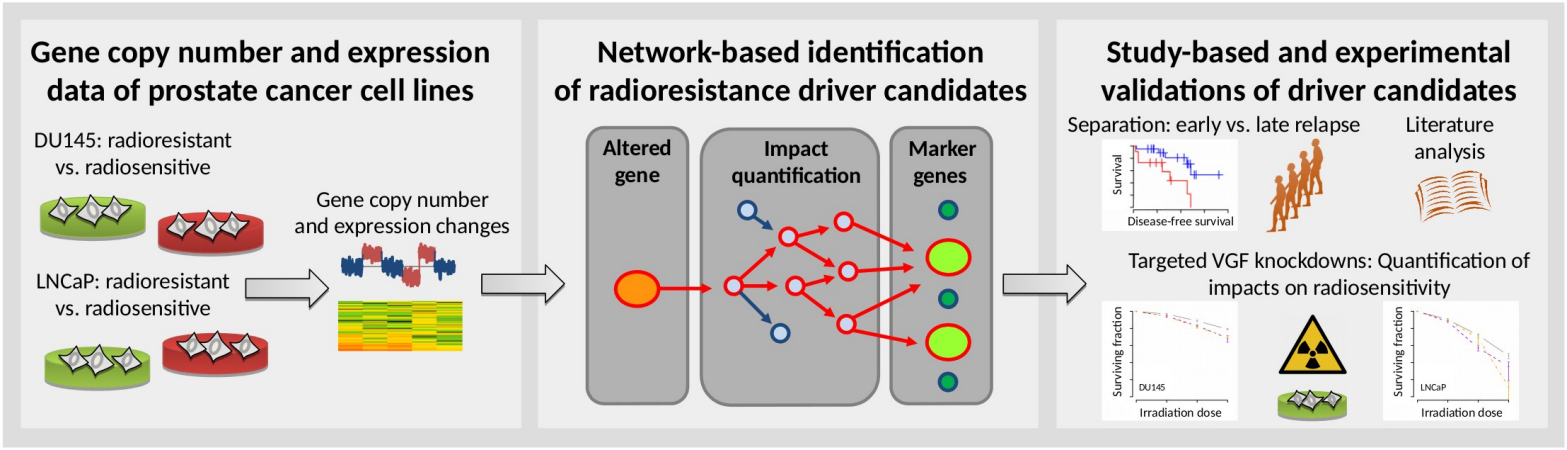
Methodological overview. Left box, Prostate cancer cell lines DU145 and LNCaP were purchased from the American Type Culture Collection and used to establish radioresistant cell lines. Gene copy number and expression profiles of radioresistant and corresponding age-matched non-irradiated radiosensitive parental cell lines were measured. Middle box, A prostate cancer-specific gene regulatory network was learned from gene expression and copy number data from 541 prostate cancer patients from The Cancer Genome Atlas (TCGA) and validated on 768 cancer cell lines of the Cancer Cell Line Encyclopedia (CCLE). This network was used to quantify putative impacts of genes with differential expression and directly underlying copy number alterations between radioresistant and radiosensitive cell lines (orange circle) on known marker genes of radioresistance (green circles) utilizing network propagation (red arrows). Right box, Identified potential radioresistance driver genes were evaluated for their potential to separate irradiated prostate cancer patients from TCGA into early and late relapse groups. In-depth literature analysis was done for all cell line-based candidate genes that were predictive for the relapse behavior of irradiated prostate cancer patients. Sophisticated experimental validations were done for the candidate gene *VGF* by analyzing the impact of siRNA-based *VGF* knockdowns on radiosensitivity. A detailed technical flow chart is shown in S1 Fig.

Here, we developed an approach for the network-based analysis of prostate cancer cell lines with acquired radioresistance to identify clinically relevant marker genes associated with radioresistance in prostate cancer patients (Fig 1). We considered the existing prostate cancer cell lines DU145 (androgen-independent with high metastatic potential derived from a brain metastasis) and LNCaP (androgen-dependent with low metastatic potential derived from a lymph node metastasis) and analyzed molecular data of radiosensitive parental cells and corresponding radioresistant cells that we had established in [33] and which we had further analyzed in [34]. We compared gene copy number and expression profiles of the radioresistant cell lines to their radiosensitive parental cells and further utilized our network-based approach to quantify the impact of differentially expressed genes with directly underlying copy number alterations on known marker genes of radioresistance. We identified several novel gene candidates that are potentially involved in the manifestation of radioresistance enabling to separate prostate cancer patients treated with radiotherapy into early and late relapse groups. We performed in-depth wet-lab validations of a selected candidate gene (*VGF*: Neurosecretory protein VGF) providing further evidence that our computational approach can contribute to the identification of genes involved in radioresistance.

## Results

### DU145 shows more gene copy number alterations than LNCaP

**Fig 2:**
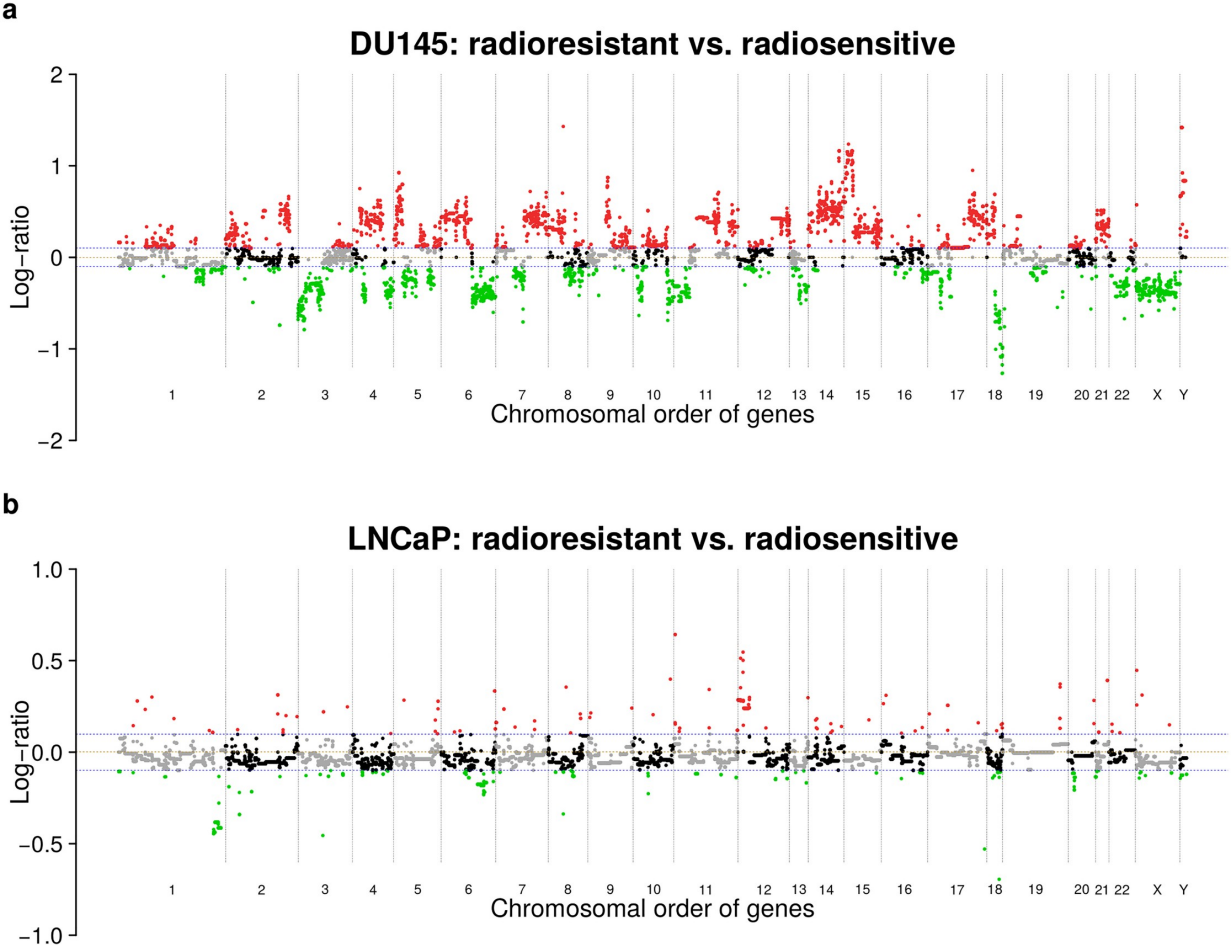
Gene copy number alterations of DU145 and LNCaP. Gene copy number profiles of DU145 (a) and LNCaP (b) comparing radioresistant to radiosensitive cell lines. Gene copy number alterations are quantified by log_2_-ratios of radioresistant versus radiosensitive and plotted in the chromosomal order of genes. Deviations of log_2_-ratios from zero (brown dashed line) indicate the presence of gene copy number alterations. Considered reduced (green dots below blue dashed line: log_2_-ratios < -0.1) or increased (red dots above blue dashed line: log_2_-ratios > 0.1) gene copy numbers in the corresponding radioresistant cell lines of DU145 and LNCaP are highlighted. Ends of chromosomes are marked by black dotted vertical lines. Unchanged genes on a chromosome are shown by alternating grey and black dots to further support the visual separation between chromosomes. An additional heatmap representation including comparisons of radioresistant and radiosensitive DU145 and LNCaP to normal reference DNA is shown in S2 Fig.

We considered radioresistant cell lines of the prostate cancer cell lines DU145 and LNCaP that were established in [33] and further characterized in [34]. We analyzed corresponding array-based comparative genomic hybridization (aCGH) experiments to identify gene copy number alterations distinguishing radioresistant DU145 and LNCaP from their radiosensitive parental cell line (Fig 1, S1 Table). Generally, radioresistant DU145 showed more copy number alterations than radioresistant LNCaP (Fig 2). In more detail, comparing radioresistant to radiosensitive DU145, 24.8% of genes (6,109 of 24,625) had reduced and 38.6% (9,498 of 24,625) had increased copy numbers (Fig 2a, S2 Table), whereas only 3.1% (765 of 24,625) of genes had reduced and 1.5% (377 of 24,625) had increased copy numbers comparing radioresistant to radiosensitive LNCaP (Fig 2b, S2 Table). For DU145, broad segments of gene copy number alterations across all chromosomes and few focal gene copy number alterations were observed (Fig 2a). In contrast, LNCaP only showed some broad regions of reduced gene copy numbers on chromosomes 1, 6, and 20, greater gene copy numbers for a broad region on chromosome 12, and some focal gene copy number alterations on different chromosomes (Fig 2b). Both cell lines further showed a significant overlap of 389 genes with reduced gene copy numbers (mainly on chromosomes 1 and 6, Fisher’s exact test: *P* = 7.16 ⋅ 10^-56^) and a non-significant overlap of 47 genes with increased copy numbers widespread across the genome.

We also compared the gene copy number alterations of radioresistant and radiosensitive DU145 and LNCaP to normal male reference DNA to better understand the observed differences between both cell lines (S2 Fig). We found that radiosensitive DU145 had much more gene copy number alterations than radiosensitive LNCaP, radioresistant DU145 and LNCaP were clearly more similar to their corresponding radiosensitive counterpart than to each other, and radioresistant DU145 had much more gene copy number alterations than radioresistant LNCaP. These findings indicate that DU145 is generally more prone to DNA copy number alterations than LNCaP, which could explain the strong differences observed between both cell lines. An increased radioresistance of DU145 in comparison to LNCaP can also contribute to these observations [33].

### DU145 and LNCaP mainly show cell line specific expression patterns

**Fig 3:**
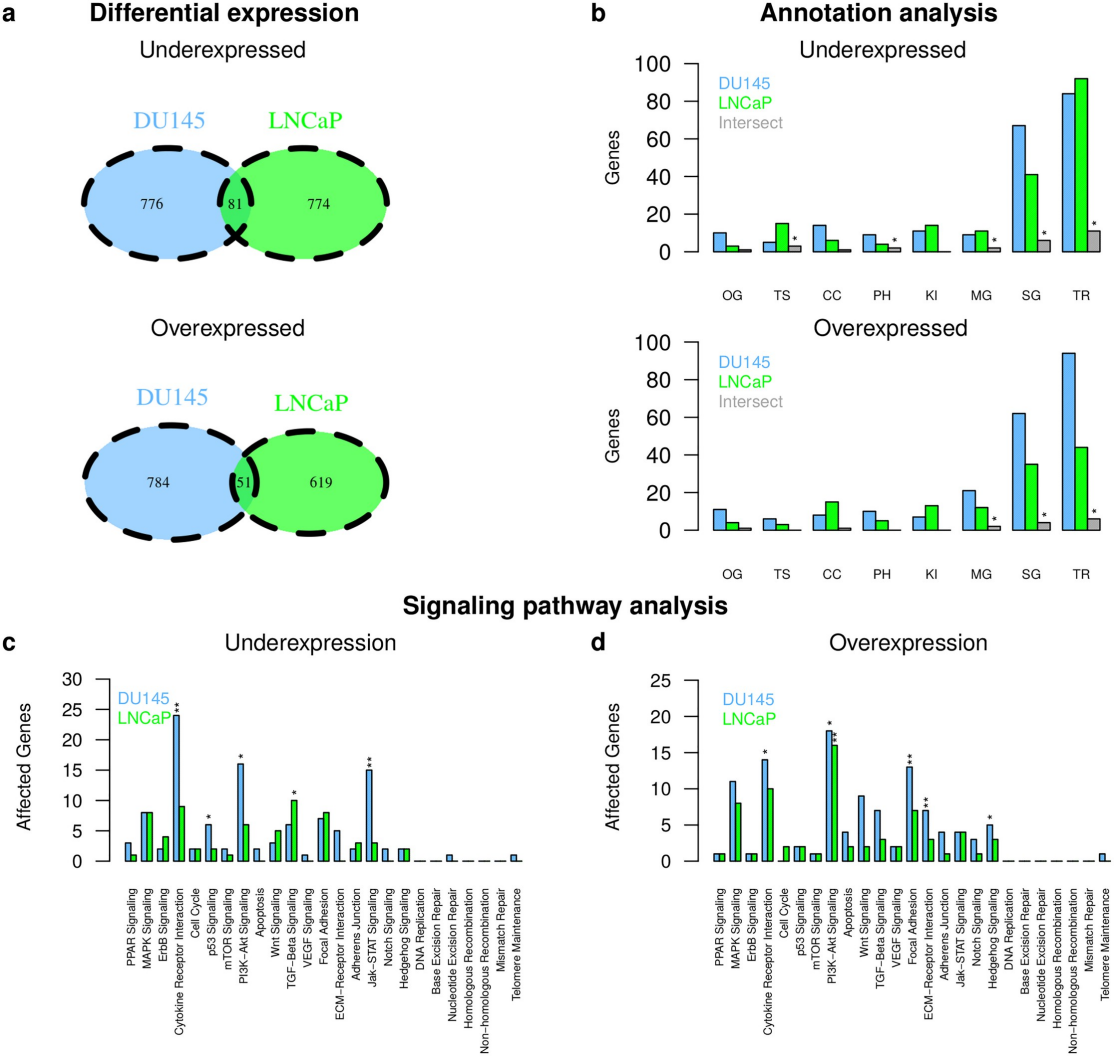
Gene expression differences between DU145 and LNCaP. Differentially expressed genes between radioresistant and radiosensitive cell lines were determined for DU145 and LNCaP. Identified under- (top panels) and overexpressed genes (bottom panels) in the radioresistant cell lines of DU145 and LNCaP were compared to each other at the single gene level (a) and at the level of cancer-relevant gene annotation categories (b; categories: oncogenes (OG), tumor suppressor genes (TS), cancer census genes (CC), phosphatases (PH), kinases (KI), metabolic pathway gene (MG), signaling pathway gene (SG), transcriptional regulator (TR)). Significant overlaps between categories are denoted by ‘*’ (b; grey columns, *P* < 0.001, Fisher’s exact test). Identified under- (c) and overexpressed genes (d) were further mapped to known cancer-relevant signaling pathways. Overrepresented pathways were highlighted by ‘*’ (*P* < 0.05, Fisher’s exact test) and ‘**’ (*P* < 0.01).

We analyzed gene expression for DU145 and LNCaP to identify differentially expressed genes between established radioresistant DU145 and LNCaP and their radiosensitive parental cell line (Fig 1). We used a Hidden Markov Model to determine differentially expressed genes [35] (see Methods). We found 857 under- and 835 overexpressed genes in radioresistant DU145 and 855 under- and 670 overexpressed genes in radioresistant LNCaP compared to their radiosensitive parental cell lines (S3 Table). The overlap of differentially expressed genes between both cell lines was small but significant (Fig 3a: 81 under- and 51 overexpressed genes, *P* < 7.46 ⋅ 10^-8^, Fisher’s exact test, S3 Table). The majority of these genes was part of signaling pathways and/or encode for transcription factors/co-factors (Fig 3b). Commonly underexpressed genes in radioresistant DU145 and LNCaP included e.g. known tumor suppressors (e.g. *BCL10*, *EPB41L4A*, *SPRED1*, *SERPINB5*) and commonly overexpressed genes included e.g. *SEMA4A* involved in cell-cell signaling and migration, *NROB1* associated with stem cell pluripotency, and genes involved in cytokine signaling (e.g. *IL19*, *IL3RA*) [36].

We found similar patterns of differential expression among known cancer-relevant signaling pathways for both cell lines (Fig 3). Further, radioresistant DU145 and LNCaP showed an enrichment of overexpressed PI3K-Akt pathway genes (Fig 3d). DU145 also showed an enrichment of underexpressed genes for the cytokine pathway, the p53 pathway, the PI3K-Akt pathway, and the Jak-STAT pathway (Fig 3c) and an enrichment of overexpressed genes for the cytokine pathway, the ECM receptor pathway, the focal adhesion pathway, and the hedgehog pathway (Fig 3d). LNCaP showed an enrichment of underexpressed TGF-Beta signaling genes (Fig 3c). Most of these pathways have already been associated with radioresistance of prostate cancer and other types of cancers (e.g. [5, 14, 37–39]).

### Direct impact of copy number alterations on expression of affected genes

We analyzed which genes with copy number alterations showed altered expression. LNCaP showed more gene expression alterations than gene copy number alterations (1,525 vs. 1,142) and only 8.9% (102 of 1,142) of genes with copy number alterations showed altered expression. 66 of these 102 genes showed putative direct impacts of the underlying copy number alteration on the expression level (S4 Table; 49 genes with reduced copy number and decreased expression; 17 genes with increased copy number and expression). These findings are similar to a related analysis of radiosensitive and radioresistant subclones of a head and neck squamous cell carcinoma cell line that only found few differentially expressed genes with directly underlying copy number alterations [40]. Further, tumor suppressor genes such as *PRDM1* and *RNF217* had a reduced copy number and showed reduced expression in radioresistant compared to radiosensitive LNCaP.

In contrast, we found substantially more gene copy number alterations than gene expression alterations for DU145 (15,607 vs. 1,692), but only 7.3% (1,144 of 15,607) of genes with altered copy numbers also showed altered expression. 447 of these 1,144 genes showed expression changes in the same direction (S4 Table; 191 genes with reduced copy number and reduced expression; 256 genes with increased copy number and increased expression), whereas the other genes had expression differences in the opposite direction possibly due to the complex genomic rearrangements observed for DU145 affecting many transcriptional regulators (Figs 2a and 3b). These findings are supported by our previous analysis of DU145 [34]. Further, tumor suppressor genes such as *EPB41L4A* and *TNFAIP3* had a reduced copy number and showed reduced expression, whereas oncogenes such as *ALDH1L2* and *WNT11* had an increased copy number and showed increased expression in radioresistant compared to radiosensitive DU145.

Generally, all genes with copy number alterations and consistent expression responses in the same direction represent putative direct driver candidates that could be involved in the manifestation of radioresistance.

### Gene copy number alterations impact on expression of known radioresistance markers

**Fig 4:**
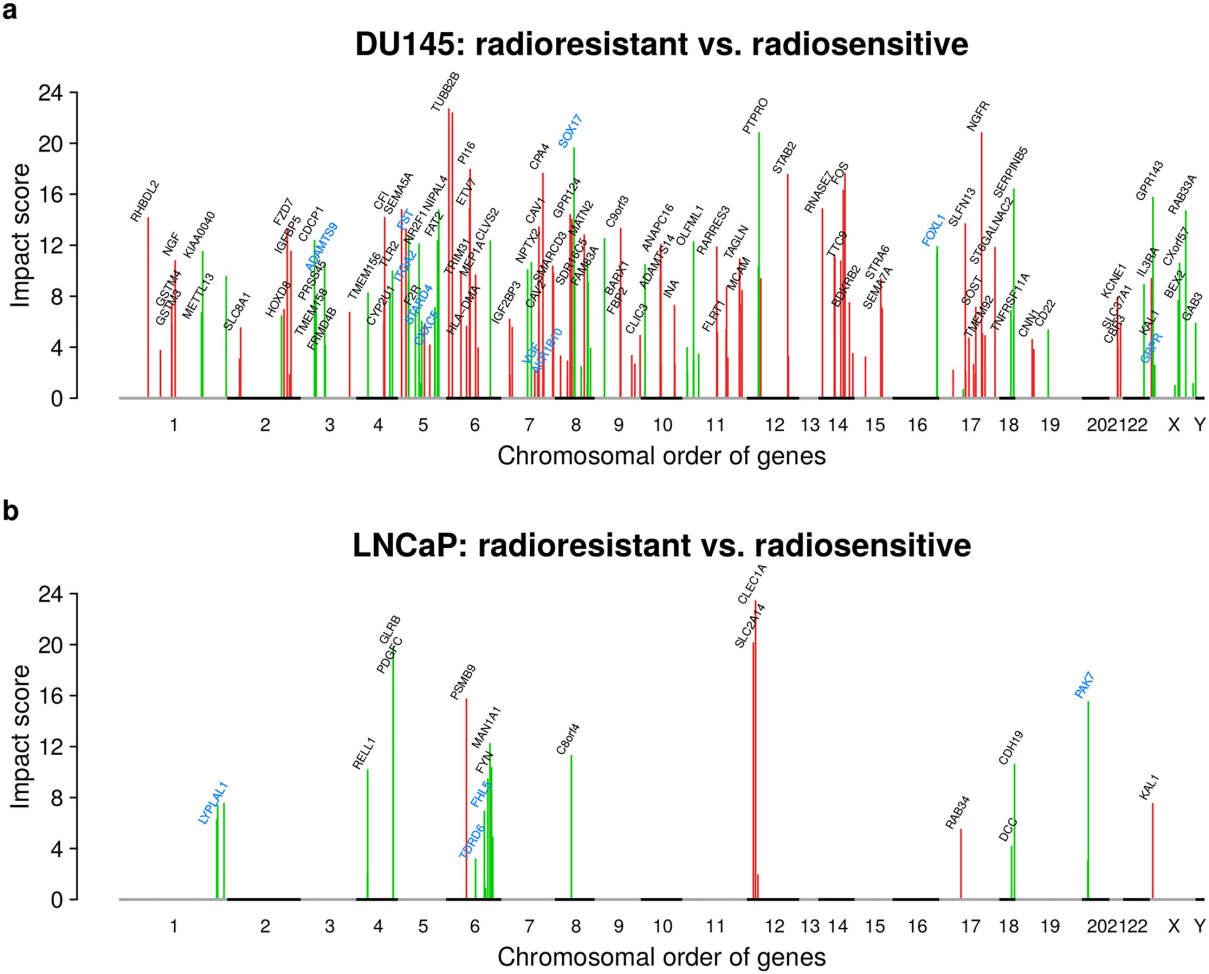
Impacts of potential radioresistance driver genes on known radioresistance markers. Impacts of differentially expressed genes with directly underlying copy number alterations in radioresistant DU145 (a) and radioresistant LNCaP (b) on known markers of radioresistance. The impact score represents the log_10_-ratio of the gene-specific impact on known radioresistance marker genes comparing the impact score reached for the prostate cancer specific network to the average impact score obtained under 10 random networks of same complexity (degree-preserving network permutations). Impact scores of genes with significantly greater impacts under the original network (*q* < 0.01) are shown by colored peaks (green: deleted and underexpressed; red: amplified and overexpressed for radioresistant vs. radiosensitive). The majority of gene names are shown. See S5 Table for names of all putative high impact genes. High impact genes that enabled a separation of TCGA prostate cancer patients into early and late relapse groups (Fig 5, S5 Fig) are highlighted in blue.

To determine which of the radioresistance driver candidates with altered expression and underlying copy number alteration putatively contribute to the manifestation of radioresistance, we computed direct and indirect impacts of these candidates on the expression of known radioresistance marker genes (Fig 1). To realize this, we first used expression and copy number data of 14,780 genes of 541 prostate cancer patients from TCGA [23] to learn a prostate cancer-specific gene regulatory network (see Methods for details). This network was able to predict expression levels of individual genes across 768 independent cancer cell lines [41] with comparable power as in a previous study with other cancer types [28] (S3 Fig). Next, we used this network to compute for each putative radioresistance driver candidate (S4 Table) its potential impact on the expression of known altered radioresistance marker genes utilizing network propagation [28, 29] (see Methods for details, Fig 1 for an illustration, and S1 Fig for a detailed work flow illustration). Putative impacts of the DU145 and LNCaP driver candidates on the expression of differentially expressed cell line specific radioresistance marker genes are shown in Fig 4.

We found 162 driver candidates for DU145 (Fig 4a) and 27 for LNCaP (Fig 4b) that strongly impact on the expression of cell line specific radioresistance markers (S5 Table, *q* < 0.01). These driver candidates comprise overexpressed genes with increased copy number and underexpressed genes with decreased copy number. Potential driver candidates were distributed across the whole DU145 genome, whereas they were more focally distributed in LNCaP (Fig 4), which is expected because of the strong differences in DNA copy number alterations between both cell lines (Fig 2).

Considering the 162 driver candidates identified from DU145 (S5 Table, Fig 4a), several overexpressed genes with increased copy numbers encode membrane proteins (e.g. *RHBDL2*, *FZD7*, *SEMA5A*, *IL7R*, *STAB2*, *GPR124*, *NGFR*, *CAV1*) and transcriptional regulators (e.g. *ETV7*, *FOS*, *ATXN1*, *LEF1*) [36]. Further, *SOX17*, a transcription factor important for embryonic development and cell fate determination, and the tumor suppressors *SEPINB5* and *PTRO* were underexpressed with underlying reduced copy number [36]. Generally, these and other driver candidates were involved in the regulation of diverse cellular processes such as cytoskeletal remodeling, cell growth, proliferation, adhesion, or migration.

Considering the 27 driver candidates identified from LNCaP (S5 Table, Fig 4b), most genes were involved in cell adhesion (underexpressed with reduced copy number: *CDH19*, *DCC*, *FERMT1*, *FYN*, *VNN2* except *CLEC1A* and *KAL1*) [36]. Again genes involved in other cancer-relevant processes such as cell proliferation, migration, differentiation, apoptosis, or cytoskeletal remodeling were among the driver candidates (all underexpressed with reduced copy number: *ARHGAP18*, *DUSP10*, *PAK7*, *PDGFC*, *RNF217*) [36]. Further, the known tumor suppressors *DCC* and *GPRC5A* were underexpressed with underlying reduced copy number.

Only *KAL1* located on chromosome X was found as common high impact gene in DU145 and LNCaP (S5 Table, Fig 4), but *KAL1* was underexpressed with reduced copy number in DU145 and overexpressed with increased copy number in LNCaP comparing radioresistant to radiosensitive cell lines. *KAL1* encodes an extracellular matrix protein involved in cell migration [36]. Downregulation of *KAL1* has been associated with increased tumor size and vascular invasion of hepatocellular carcinoma [42]. Similarly, silencing of KAL1 squamous cell carcinoma accelerated the G1 to M phase transition promoting cell proliferation and colony formation [43].

Generally, the small overlap between DU145 and LNCaP was expected due to strongly different copy number alteration profiles (Fig 2). Still, both sets of cell line specific driver candidates tend to act on the same cellular processes that could contribute to the manifestation of radioresistance.

### Potential radioresistance drivers separate irradiated patients into early and late relapse groups

Next, we tested which of the identified cell line specific radioresistance driver gene candidates could potentially be relevant to predict the relapse behavior of prostate cancer patients treated by radiation therapy. To realize this, we analyzed the expression behavior of the driver candidates within a subgroup of 32 irradiated prostate cancer patients available from TCGA [23] (S6 Table). To enable relapse predictions for patients, only driver candidates with consistent expression behavior between radioresistant cell lines and irradiated patients were considered. Thus, a driver candidate that was underexpressed in radioresistant DU145 or LNCaP shows consistent behavior when irradiated patients with low expression of this gene tend to show faster relapses than patients with higher expression. In analogy, a driver candidate that was overexpressed in radioresistant DU145 or LNCaP shows consistent behavior if irradiated patients with high candidate gene expression tend to show faster relapses than patients with lower expression. We applied this consistency filtering to all driver candidates by comparing the cell line specific candidate gene expression behavior to the corresponding correlation between candidate expression and time until relapse in patients (see Methods for details). We found that 61 of 162 candidates from DU145 and 14 of 27 from LNCaP showed consistent expression behavior between cell lines and irradiated patients (S5 Table).

**Fig 5:**
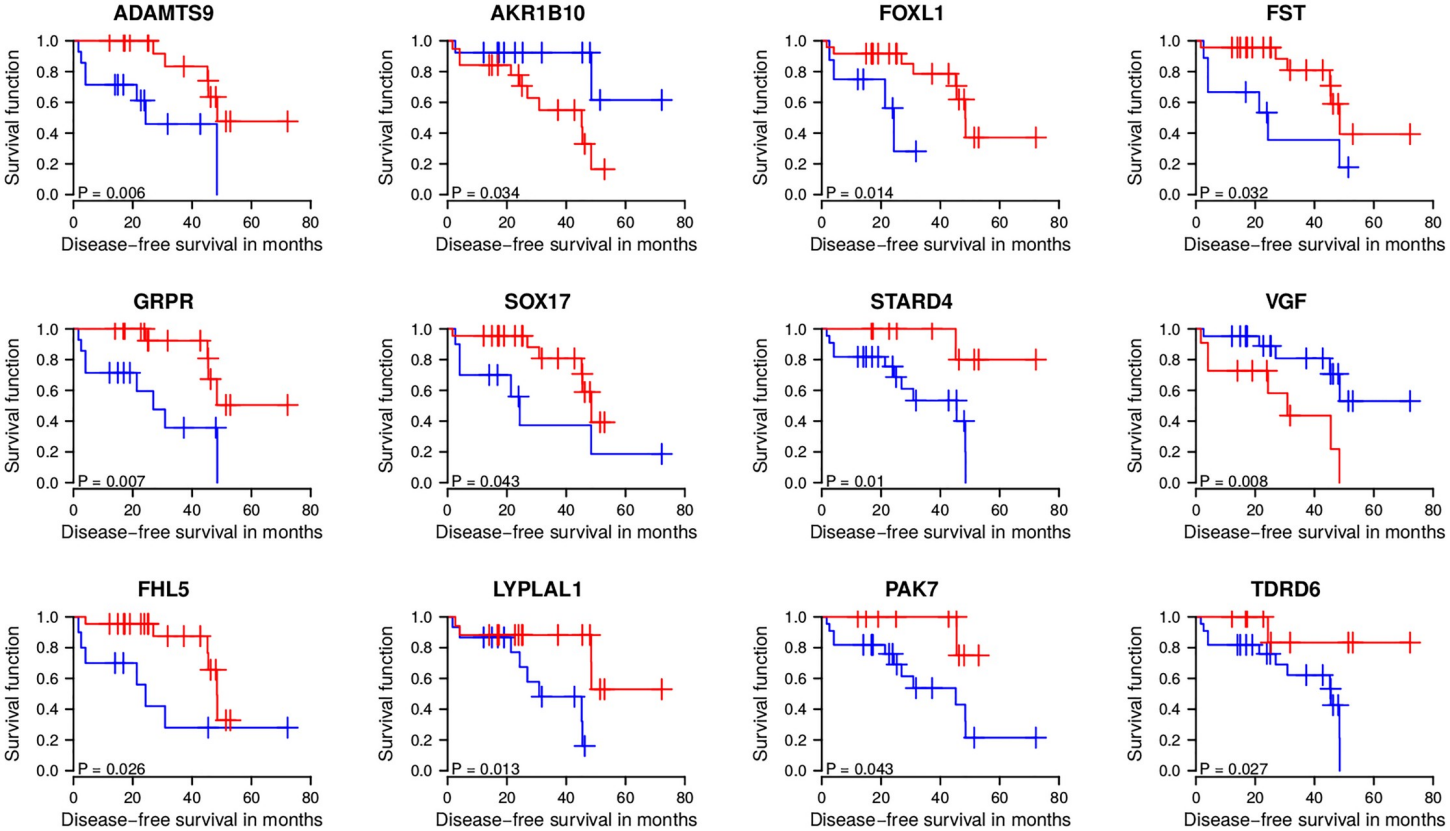
Marker gene-based separation of irradiated prostate cancer patients into early and late relapse groups. Potential radioresistance driver genes revealed from DU145 (top and middle row) and LNCaP (bottom row) were analyzed for their expression behavior in 32 irradiated prostate cancer patients from TCGA. Expression levels of each marker gene across the 32 patients were used to determine a marker gene-specific optimal cutoff for disease-free survival risk curves separating patients with low (blue curve) and high (red curve) marker gene expression with respect to the constraint that at least 8 patients must be assigned to each curve. Log-rank test p-values indicate that these selected marker genes enable a separation of irradiated prostate cancer patients into early and late relapse groups. Shown are standard approximate log-rank test p-values that only marginally deviated from exact log-rank p-values determined by exhaustive computations, except for FOXL1 that had a clearly less significant exact log-rank p-value of 0.076 (see Methods for details and S4 Fig). See S1 Text for a detailed discussion of the driver candidates in the context of the existing literature.

Next, we analyzed each of these candidate genes for its potential to distinguish between early and late relapse of irradiated prostate cancer patients by performing a Kaplan-Meier analysis. Under consideration that the early or late relapse group must contain at least eight patients, we found that 10 of 61 driver candidates from DU145 and 4 of 14 from LNCaP have the potential to distinguish between early and late relapse (S5 Table, Log-rank tests: *P* < 0.05 and corresponding conservative false discovery rates estimated between 14% and 22% [44] and more liberal estimates between 3% and 5% [45]). We also analyzed if the standard log-rank p-value computation for our small cohort of 32 patients with its determined different-sized early and late relapse subgroups had led to biased p-values [46]. We therefore computed the exact permutational log-rank p-values with the ExaLT method [46] for all DU145 and LNCaP driver candidates and compared them to the corresponding approximate log-rank p-values of our initial standard analysis. We found that the approximate log-rank p-values mostly overestimated the significance of the marker candidates, but this only marginally affected the ten driver candidates from DU145 (except for *FOXL1*: log-rank p-value increased from 0.014 to 0.076) and the four driver candidates from LNCaP and was clearly more pronounced for larger insignificant p-values (S4 Fig). The selected driver candidates are shown in Fig 5 and S5 Fig and listed in Table 1. Corresponding copy number alteration levels are shown in S6 Fig.

**Table 1:** Summary of potential radioresistance drivers.

Gene	Faster Relapse	Annotations
ADAMTS9	low expression	protease function, renal tumors
AKR1B10	high expression	all-trans-retinaldehyde reductase, detoxification
FOXL1	low expression	transcription factor, proliferation, differentiation, metabolism
FST	low expression	follistatin, sexual hormone
GRPR	low expression	receptor for gastrin releasing peptide, associated with activation of phosphatidylinositol messenger system
SOX17	low expression	transcription factor, inhibits Wnt signaling, key regulator of embryonic development
STARD4	low expression	putative role in intracellular transport of sterols and other lipids
VGF	high expression	nerve growth factor inducible protein, regulation of cell-cell interactions
FHL5	low expression	putative role in spermatogenesis, stimulates CREM activity
LYPLAL1	low expression	lysophospholipase like 1, no phopholipase activity, able to hydrolyze short chain substrates
PAK7	low expression	protein kinase, involved in cytoskeleton regulation, cell migration, cell proliferation, and cell survival
TDRD6	low expression	involed in spermatogenesis, chromatin body formation, miRNA expression
CXXC5	high expression	required for DNA-damage induced phosphorylation, p53 activation and cell cycle arrest
ITGA2	low expression	trans-membrane receptor subunit, cell adhesion

Potential driver genes of radioresistance dividing irradiated prostate cancer patients from TCGA into early and late relapse groups. The column ‘Faster Relapse’ reports if patients with low or high gene-specific expression levels showed a faster relapse in the corresponding Kaplan-Meier curves shown in Fig 5 and S5 Fig. See S1 Text for a detailed discussion of the driver candidates in the context of the existing literature.

We found that high expression of *AKR1B10* or *VGF* was associated with patients that had a faster relapse than patients with lower expression of these genes (Fig 5). Further, low expression of *ADAMTS9*, *FOXL1*, *FST*, *GRPR*, *SOX17*, *STARD4*, *FHL5*, *LYPLAL1*, *PAK7*, *TDRD6*, *CXXC5*, or *ITGA2* was associated with patients that showed a faster relapse than patients with corresponding higher expression levels (Fig 5, S5 Fig). A detailed discussion of the identified driver candidates in the context of the existing literature is provided in S1 Text. Since patient-specific expression profiles were measured before radiation, theses driver candidates potentially represent markers whose expression behavior may allow to decide if a prostate cancer patient would profit from a radiation therapy or not.

Further, we investigated if the disease status after initial treatment of irradiated patients had biased the observed separations into early and late relapse groups, but we did not find any significant difference with respect to the distribution of patients with complete and non-complete remission after initial treatment between both groups (Fisher’s exact tests: *P* ranged from 0.69 to 1).

Finally, we analyzed how our predicted driver candidates contribute to the modeling of the disease-free survival in the presence of additional covariates. Therefore, we used Cox regression [47, 48] to determine the contribution of prognostic factors (age, clinical T-stage, Gleason score, psa) with and without considering group assignments based on each driver candidate. We found that the prognostic factors alone were not well-suited to model the disease-free survival, whereas the driver candidates provided important information for the modeling of the disease-free survival in the presence of the other factors (S7 Fig).

### *VGF* and *FHL5* also tend to predict relapse behavior of non-irradiated patients

Some of these marker candidates might also be associated with relapse of prostate cancer independent of radiation therapy. We therefore further analyzed the expression behavior of the marker candidates for 182 prostate cancer patients from TCGA that did not receive an adjuvant radiation therapy (S6 Table) [23]. We again tried to group the patients into early and late relapse groups by performing a Kaplan-Meier analysis using the same driver gene-specific expression cutoffs determined in the prior analysis. We found that only *VGF* and *FHL5* enabled a similar separation of non-irradiated patients as observed for irradiated patients (S5 Fig, Log-rank test: *P* < 0.1). As for irradiated patients (Fig 5), high expression of *VGF* was associated with early relapse, whereas high expression of *FHL5* was associated with late relapse of non-irradiated patients (S5 Fig). Thus, both marker genes may also have at least some general prognostic potential for relapse, but only the increased expression of *VGF* in early relapse patients is of greater potential therapeutic relevance, because knockdowns are potentially better to realize than knockins.

### Validation of *VGF* by *in vitro* radiobiological assays

We selected the neuroendocrine factor *VGF* for in-depth validation studies. This was motivated by our observation that *VGF* showed increased expression in prostate cancer patients with early relapse (Fig 5) and further triggered by recent studies that highlighted the importance of *VGF* in different types of cancer [49–52].

**Fig 6:**
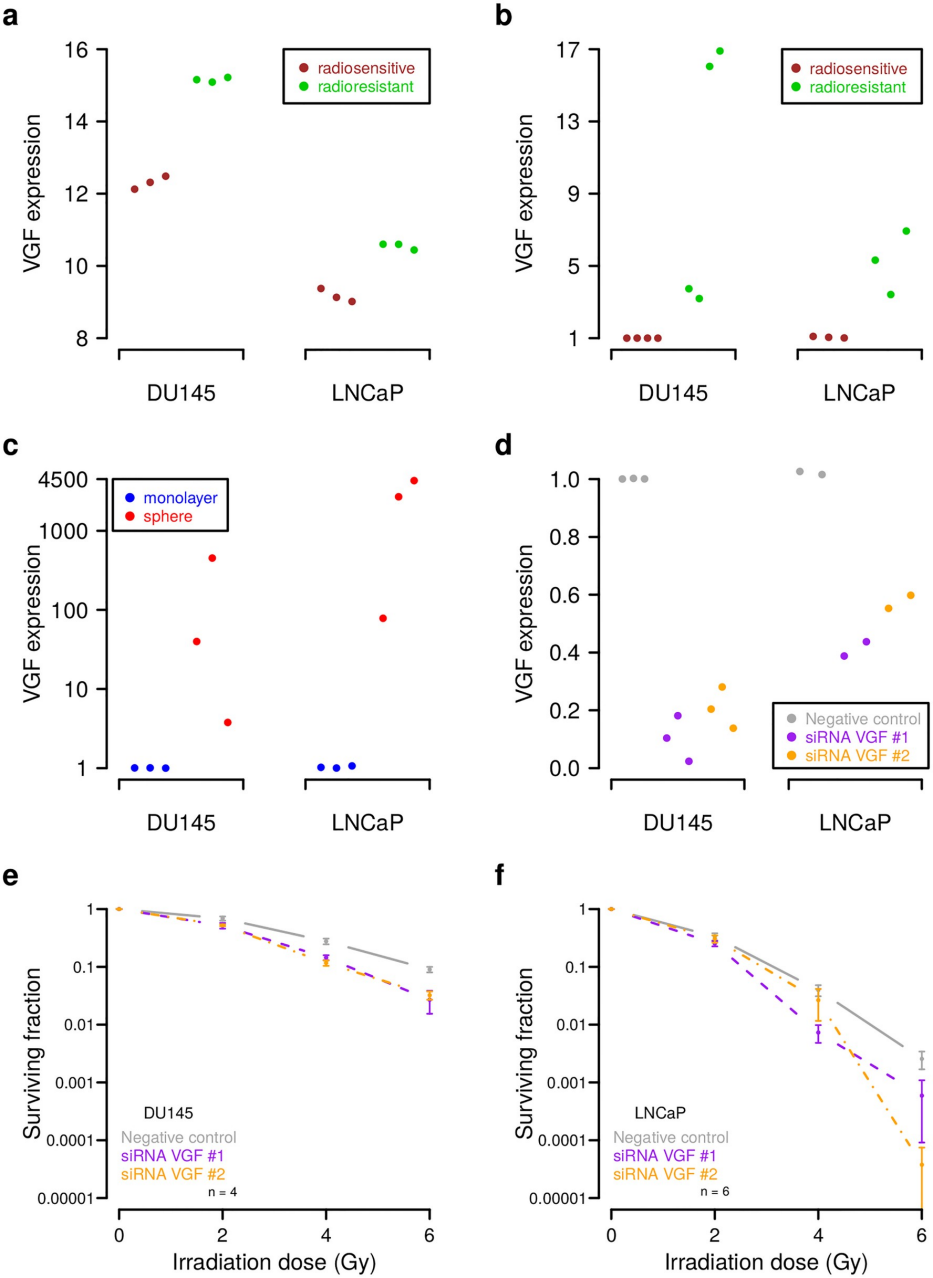
Experimental validation of *VGF* as regulator of cell radioresistance. (a) Increased *VGF* expression in radioresistant DU145 and LNCaP in comparison to their radiosensitive parental cell lines in our microarray data. Three biological replicates were considered for each condition. (b-d) RT-qPCR analysis of *VGF* expression under different conditions. (b) Increased *VGF* expression in four independent radioresistant DU145 and three independent radioresistant LNCaP clones relative to their radiosensitive parental cell lines. (c) Increased *VGF* expression in sphere relative to monolayer cultures of parental DU145 and LNCaP cells. (d) Reduced *VGF* expression in parental DU145 and LNCaP cells induced by siRNA-mediated gene silencing relative to negative controls. (e-f) Increased radiosensitivity of parental DU145 and LNCaP cells induced by siRNA mediated reduction of *VGF* expression. Shown are average fractions of surviving cells in log_10_-scale for increasing radiation dose. Error bars represent the standard error of the mean and ‘n’ specifies the number of biological replicates. Corresponding linear-quadratic (LQ) model curves are shown in S11 Fig.

We found that *VGF* was significantly upregulated in DU145 and LNCaP prostate cancer radioresistant cell lines in our genome-wide gene expression analysis (Fig 6a; average expression difference of 2.85 in DU145 and 1.37 in LNCaP, t-tests: *P* < 0.01, S7 Table). We further analyzed the expression of *VGF* in independent radioresistant clones of DU145 and LNCaP in comparison to their radiosensitive parental cell lines and found that *VGF* was also significantly overexpressed in these clones (Fig 6b, t-test: *P* < 0.05 for DU145 and *P* < 0.03 for LNCaP, S7 Table). Interestingly, two of the four radioresistant DU145 clones had *VGF* expression levels that were comparable to those of the radioresistant LNCaP clones. These two radioresistant DU145 clones may not have an increased *VGF* copy number, but they still showed significantly increased *VGF* expression in comparison to the parental radiosensitive DU145 cell line (t-test: *P* < 0.04). This is comparable to the overexpression of *VGF* in radioresistant LNCaP without a directly underlying *VGF* copy number alteration and supports that increased *VGF* expression could contribute to increased radioresistance.

We further analyzed the expression behavior of *VGF* in parental DU145 and LNCaP cells grown under sphere-forming conditions (see S8 Fig for microscope images) that enrich cancer stem cell populations [53]. This was motivated by a recent study that showed that *VGF* is an important regulator of glioma stem cells [52]. We found that *VGF* expression was strongly increased under sphere-forming compared to monolayer conditions (Fig 6c, t-tests: *P* < 0.06 for DU145 and *P* < 0.02 for LNCaP, S7 Table). This observation was also supported by an additional analysis of the prostate cancer cell line PC3 that showed a moderately increased *VGF* expression under sphere-forming conditions (S8 and S9 Figs, t-test: *P* < 0.02, S7 Table).

Next, we considered the parental DU145 and LNCaP cells to determine changes in their radiosensitivity in response to reduced *VGF* expression by siRNA-mediated gene silencing. We first validated the knockdown efficiency by RT-qPCR and found clearly reduced *VGF* expression in *VGF* knockdowns compared to negative controls in both cell lines, where the efficiency was greater for DU145 than for LNCaP (Fig 6d, t-tests: *P* < 0.002 for DU145 and *P* < 0.02 for LNCaP, S7 Table). We also tried to validate the *VGF* knockdown by Western blots, but the two tested *VGF* antibodies (anti-VGF Santa Cruz sc-365397, B-8 mouse; St. John’s Laboratory, STJ96661, rabbit, polyclonal) gave unspecific bands that were not consistent with the corresponding RT-qPCR data (S10 Fig). Since we had confirmed *VGF* knockdowns by RT-qPCR (Fig 6d), we next performed clonogenic assays to analyze the impact of *VGF* knockdowns on radiosensitivity. We found that an inhibition of *VGF* significantly increased the radiosensitivity of DU145 and LNCaP in comparison to controls transfected with scrambled siRNAs (Fig 6e and 6f; e.g. t-tests: *P* < 0.02 for siRNA VGF #2 vs. negative control at 4 Gy for DU145 and LNCaP, S7 Table). In addition, clearly lower surviving fractions of LNCaP cells further suggest that DU145 is more radioresistant than LNCaP, which is in accordance with our prior findings [33, 34].

Finally, we also considered the prostate cancer cell line PC3 and could confirm the efficiency of *VGF* knockdowns and we also observed a moderately increased radiosensitivity in response to *VGF* knockdowns (S9 Fig, e.g. t-test: siRNA VGF #2 vs. negative control: *P* < 0.03 at 4 Gy, S7 Table). We further estimated linear-quadratic models [54] of the clonogenic survival data of DU145, LNCaP, and PC3 to obtain functional representations of the individual survival curves (S11 Fig).

## Discussion

Radioresistance of prostate cancer is driven by different cellular processes enabling cancer cells to survive radiation doses that can safely be delivered to the tumor [4, 5]. Molecular markers are urgently needed to better predict the clinical outcome of radiotherapies and to develop targeted adjuvant strategies to sensitize radioresistant cells. Radioresistant prostate cancer cell lines represent an important model system for the identification of novel candidate genes and the analysis of molecular mechanisms involved in radioresistance, but they typically show large chromosomal deletions and amplifications that affect many genes. This in combination with the small number of cell lines that are usually profiled and their cell line specific gene copy number and expression profiles does not allow a straightforward identification of radioresistance drivers by standard statistical approaches for gene copy number and expression analysis. In this situation, it is almost impossible to derive promising candidates from hundreds or thousands of differentially expressed genes with directly underlying gene copy number alterations without prior knowledge about genes involved in altered cellular processes that contribute to radioresistance.

Therefore, we developed a network-based method to jointly analyze the gene copy number and expression profiles of an individual cell line to distinguish potential drivers from passengers. The essential basis of this approach was the prostate cancer-specific gene regulatory network that we learned from gene expression and copy number data of 541 prostate cancer patients from TCGA. This network inference was very time and resource consuming requiring 670 hours on a high-performance compute server (Taurus ZIH TUD). Validations on data of 768 cancer cell lines from [28, 41] confirmed that this network can predict the expression behavior of individual genes in cancer cell lines enabling an analysis of the prostate cancer cell lines DU145 and LNCaP. This analysis is limited by the fact that the cancer samples from TCGA and our cell lines were analyzed on different experimental platforms. Both data sets also showed differences in the number of expressed genes, where more genes were expressed in our cell line models than in the cancer samples. Thus, it is clear that not all observations form our *in vitro* prostate cancer cell lines are transferable to the *in vivo* situation in prostate tumors. Nevertheless, we applied network propagation to differentially expressed genes with directly underlying copy number alterations from DU145 and LNCaP to determine their impacts on known markers of radioresistance. Comparisons to random networks of same complexity (degree-preserving network permutations) in combination with further filtering revealed ten candidates from DU145 (*ADAMTS9*, *AKR1B10*, *CXXC5*, *FST*, *FOXL1*, *GRPR*, *ITGA2*, *SOX17*, *STARD4*, *VGF*) and four from LNCaP (*FHL5*, *LYPLAL1*, *PAK7*, *TDRD6*) that were able to distinguish irradiated prostate cancer patients from TCGA into early and late relapse groups. A detailed discussion of these candidate genes is given in S1 Text. These candidate genes may allow to develop biomarkers for the analysis of biopsy samples to predict relapse risk and to adapt treatment for individual prostate cancer patients. Targeted perturbations of these genes may allow to increase the radiosensitivity of prostate cancer cells. Additional preclinical and clinical studies are required to validate these candidates.

We experimentally validated the novel radioresistance marker gene candidate *VGF*, a neuroendocrine factor, that was highly overexpressed in DU145 and LNCaP radioresistant prostate cancer cell lines and whose high expression was associated with shorter disease-free survival of irradiated prostate cancer patients. *VGF* was originally identified in a pheochromocytoma cell line in response to the addition of the nerve growth factor (NGF) [55]. *VGF* is an important regulator of metabolism and endoplasmic reticulum (ER) stress in neurons and endocrine cells [56–58], where it activates pro-survival signaling pathways such as PI3K/AKT/mTOR and MAPK/ERK1/2 [59, 60], but its role in regulation of cancer cells remained unclear for a long time. Experimental evidences from *in vitro* models, mouse xenografts and analysis of patient outcomes showed that *VGF* expression is associated with resistance to EGFR inhibitors and further induces epithelial-mesenchymal transition (EMT) and tumor cell dissemination [50, 51]. In addition, *VGF* has been shown to be preferentially expressed in glioblastoma stem cells promoting glioblastoma stem cell survival and stemness and to further support survival of differentiated glioblastoma cells to promote tumor growth [52]. Our previous studies showed that the emergence of radioresistance also triggers EMT, increases migratory properties, and further results in enrichment of cancer stem cell populations in prostate cancer cells [33]. In accordance with this, our *in vitro* validation experiments confirmed an upregulation of *VGF* expression in additionally analyzed independent radioresistant DU145 and LNCaP clones, showed that *VGF* is highly expressed under sphere forming conditions, and further demonstrated that *VGF* knockdowns lead to increased radiosensitivity. These results suggest that VGF is involved in radioresistance of prostate cancer. This is also supported by our findings for the prostate cancer cell line PC3.

For Western blotting analysis of *VGF* in response to siRNA-mediated gene silencing, we tried two available antibodies (anti-VGF Santa Cruz sc-365397, B-8 mouse; St. John’s Laboratory, STJ96661, rabbit, polyclonal) and we additionally performed RT-qPCR analysis of *VGF* expression as control in parallel. Although we observed a pronounced knockdown of *VGF* by RT-qPCR, we did not observe the specific *VGF* band by Western blotting, which can be explained by the observation of substantial background signals. Therefore, we focused on PCR-based analysis of VGF expression in our validation studies.

We observed that VGF knockdowns were more efficient in DU145 than in LNCaP, but fewer LNCaP cells survived irradiation. The relation between knockdown efficiency and cell survival after irradiation is complex. Different factors can contribute to cell line specific radioresistance. We already know from our prior studies [33, 34] that DU145 is more radioresistant than LNCaP. This is in accordance with our observation that DU145 had substantially more DNA copy number alterations than LNCaP and could explain better survival of DU145 cells in response to irradiation by a greater tolerance of DNA double strand breaks. Further, the knockdown efficiency also depends on the protein turnover rate [61] and highly expressed genes can be more susceptible to siRNA-mediated gene silencing [62]. Thus, the found stronger expression of *VGF* in DU145 than in LNCaP may also have influenced the *VGF* knockdown efficiency observed for both cell lines. Nevertheless, our clonogenic assays clearly indicate that *VGF* could be involved in the regulation of radioresistance.

Further, our *in vitro* characterization of DU145 and LNCaP is limited to the identification of molecular alterations that are associated with intrinsic cellular radioresistance. Additional preclinical and clinical studies are necessary to further analyze the revealed marker genes in *in vivo* studies. Especially the tumor microenvironment and immune signatures of tumors can be altered by radiation therapies influencing tumor progression and therapy response [6–12]. Thus, also microenvironmental and immunomodulatory factors, which we could not cover by our analysis, can strongly influence the response of individual tumors to radiation therapy. Such and other limitations of *in vitro* cancer models have been reported over the last years [63] and special care has to be taken on work with cancer cell lines [64]. For example, in a transgenic breast cancer model tumors with similar growth characteristics but different immune signatures differed in their response to radiation therapy [65]. Therefore, a combination of radiation and immune therapy is important to improve patient outcomes [11, 66]. Another example is the treatment of the prostate cancer cell line PC3 with the HIV protease inhibitor nelfinavir that resulted in a small but significant increase of radiosensitivity *in vitro* which was not observed in corresponding PC3 xenografts [67]. Still, our analysis of revealed markers that distinguished between early and late relapse of irradiated prostate cancer patients provides a first important hint that these markers have the potential to enable predictions for the *in vivo* situation.

In summary, our detailed literature analysis and results of radiobiological assays for the maker gene *VGF* suggest that our network-based approach can predict potentially clinically relevant driver candidates involved in radioresistance of prostate cancer.

## Materials and methods

A detailed flow chart of our developed data analysis pipeline is shown in S1 Fig. See Fig 1 for a high-level overview.

### Identification of gene copy number alterations

Array-based comparative genomic hybridization (aCGH) was used to compare the genomes of radioresistant to radiosensitive cell lines for DU145 and LNCaP and to compare these genomes to normal reference DNA (Agilent Euro Male). Experiments were done on Agilent’s SurePrint G3 Human CGH Microarray Kit 2x400K (Design ID: 028081, Agilent) and performed and standardized as described in [68]. Normalized measurements were used to compute aCGH profiles. An aCGH profile represents for each of the 294,371 genomic probes a log_2_-ratio that compares the probe-specific DNA copy number in a radioresistant cell line relative to its radiosensitive counterpart (or to compare DNA copy numbers of a radioresistant or radiosensitive cell line to normal DNA). aCGH profiles were sorted according to chromosomal locations of probes and further segmented into chromosomal regions of constant copy number using DNAcopy [69]. Corresponding DNA segmentation profiles are provided in S1 Table. Copy number values of 24,625 genes (focusing on genes for which we also measured expression) were determined by mapping chromosomal locations of genes to the aCGH segments as described in [28]. The resulting log_2_-ratio gene copy number values were used to determine genes with increased or reduced copy number in radioresistant DU145 or LNCaP relative to their non-resistant counterpart using an absolute log_2_-ratio cutoff of 0.1 (Fig 2, S2 Table). The choice of this cutoff was motivated by moderately increased or decreased gene copy number alteration values comparing radioresistant to radiosensitive LNCaP. This choice did not influence the network inference and the computation of the network propagation matrix. This cutoff only defines a filter for the selection of candidate genes that were considered for more in-depth analyses. A heatmap representation that summarizes all gene copy number comparisons is shown in S2 Fig. aCGH data have been deposited in the Gene Expression Omnibus (GEO) database, accession no GSE134500.

### Identification of differentially expressed genes

Gene expression levels of radioresistant and radiosensitive cell lines of DU145 and LNCaP were measured in three biological replicates. Experiments were done on Agilent’s SurePrint G3 Human Gene Expression 8x60K v2 microarrays (Design ID: 039494, Agilent) and performed as described in [34]. Hybridization signals of 24,625 genes of all cell line specific experiments were quantile normalized [70]. Expression differences between the three radioresistant and the three radiosensitive LNCaP cell lines were not strong enough to enable a prediction of differentially expressed genes by standard t-tests with significant p-values after correction for multiple testing. Still, the t-test statistic, the p-value or the average log-ratio provide important information to rank genes according to their expression differences. We therefore used a specifically designed three-state Hidden Markov Model (HMM) to identify differentially expressed genes [35]. We trained two independent HMMs, one for DU145 and one for LNCaP, on the average gene expression log_2_-ratio profile comparing radioresistant to radiosensitive cell lines to account for cell line specific expression characteristics. This training was done with standard settings and initial state-specific means of -1.25 (underexpressed), 0 (unchanged), and 1.25 (overexpressed). We used state-posterior decoding to assign each gene to its most likely underlying state (underexpressed, unchanged, or overexpressed) in radioresistant relative to radiosensitive cell lines (S3 Table). Gene expression data have been deposited in the Gene Expression Omnibus (GEO) database, accession no GSE134500.

### Inference of prostate cancer-specific gene regulatory network

We learned a prostate cancer-specific gene regulatory network to predict potential impacts of gene copy number alterations in DU145 and LNCaP on known radioresistance marker genes. We downloaded aCGH profiles and gene expression data of 541 prostate cancer patients from TCGA and processed them as described in [28]. In addition, we removed all genes with very low constant or nearly non-variable expression across all patients and only kept genes with on average at least 1 transcription unit (normalized RSEM [71] counts from TCGA) per patient. Gene copy number and expression measurements of the remaining 14,780 genes were used to learn a gene regulatory network as outlined in [28] using the R package regNet [29]. Briefly, the expression of a specific gene was modeled as a linear combination of its copy number and the expression of all other genes. Lasso regression [72] in combination with cross validation and a significance test for lasso [73] were used to determine for each gene those predictors (e.g. gene-specific copy number or expression levels of other genes) that best explained the expression behavior of the considered gene across all prostate cancer patients. As done in [28], we focused on the most relevant links (p-values approximately zero) and removed spurious local regulators (local gene cutoff of 50) resulting in a prostate cancer-specific network with 60,447 activator and 2,105 inhibitor links between genes (S8 Table). We further confirmed that this network was capable to predict the expression of genes in cancer cell lines outperforming random networks of same complexity derived by degree-preserving network permutations (S3 Fig). The predictive power of our network was comparable to the predictive power of other networks that we had learned with the same lasso approach [28].

### Impact quantification of gene copy number alterations on radioresistance markers

We applied network propagation [28] in combination with the prostate cancer-specific gene regulatory network to determine impacts of differentially expressed genes with underlying gene copy number alterations on the expression of known radioresistance marker genes. We used the R package regNet [29] to compute a specific impact matrix based on the cell line specific log-ratio gene copy number and expression profiles comparing the radioresistant cell line to its radiosensitive counterpart for DU145 and LNCaP separately. Each cell line specific impact matrix quantifies for each gene pair (*a*, *b*) how strong gene *a* acts on the expression of gene *b* by computing the impact that flows from gene *a* to gene *b* via all possible network paths in the prostate cancer-specific gene regulatory network connecting both genes under consideration of the predictive power of individual genes. More weight was given to genes with greater positive correlations than to genes with smaller positive correlations utilizing gene-specific correlation estimates obtained from cancer cell lines (S3 Fig). Next, we only considered potential radioresistance driver candidates focusing on genes with increased expression and underlying increased copy number and on genes with decreased expression and underlying decreased copy number in radioresistant versus radiosensitive cell lines (S4 Table). In total, 292 of 447 genes for DU145 and 40 of 66 genes for LNCaP that fulfilled these criteria were also expressed in prostate cancer samples of TCGA patients. We considered each candidate gene and determined its average impact on known differentially expressed cell line specific radioresistance markers (DU145: *CCL2*, *CLDN4*, *MRC2*, *SNAI2* overexpression; LNCaP: *CXCR4* underexpression in radioresistant vs. radiosensitive cell lines; S3 Table) from the cell line specific impact matrix (S5 Table). Finally, we determined which of the potential radioresistance driver candidates had significant impacts on these differentially expressed cell line specific radioresistance markers. Therefore, we computed corresponding average impacts under 10 random networks of same complexity as the original prostate cancer-specific network. These random networks were derived based on degree-preserving network permutations by exchanging active predictors between gene-specific linear models while keeping the number of incoming and outgoing links constant for each gene. We compared the driver gene specific random impacts to the corresponding original impact by computing differences between the original impact and each corresponding random impact and used t-tests to determine which gene-specific differences in impact scores were significantly greater than zero. Genes with impacts significantly greater than under random networks were selected based on FDR-adjusted p-values (q-values) with *q* < 0.01 [44] leading to 162 potential driver candidates for DU145 and 27 for LNCaP (S5 Table).

### Transfer of cell line specific radioresistance driver genes to prostate cancer patients

We analyzed the expression of potential radioresistance drivers of DU145 and LNCaP in prostate cancer patients from TCGA to identify marker candidates that distinguish between early and late relapse after adjuvant radiation therapy. Sufficient meta-information about initial treatment, treatment response and disease free survival were available for 214 of 541 prostate cancer patients of which 32 patients received radiation and 182 did not (S6 Table). All patients were either disease free or showed a relapse after initial treatment. In more detail, the majority of patients showed a complete remission (156 of 214), whereas other patients showed a stable disease (22 of 214), partial remission (23 of 214), or a progressive disease (13 of 214) after initial treatment. To determine marker genes that distinguish between early and late relapse, only genes with consistent expression behavior between radioresistant cell lines and irradiated patients were considered. Therefore, we translated the observed expression state of a potential marker candidate from the cell lines into a meaningful interpretation for irradiated tumor patients. We assumed that if a marker candidate was overexpressed (underexpressed) in the radioresistant compared to the radiosensitive cell line, then this overexpression (underexpression) may contribute to radioresistance. Consequently, irradiated patients with high (low) expression levels of this gene may show a faster relapse than irradiated patients with lower (higher) expression levels. Thus, a negative (positive) correlation between marker gene-specific expression in patients and disease free survival is expected.

To realize this, we first considered gene expression profiles of tumors before treatment to compute correlations between the expression of each potential radioresistance driver gene and the months until relapse (disease free survival) considering all 12 of 32 irradiated patients that had a relapse (S6 Table). Next, we compared the obtained gene specific correlations to the corresponding expression states observed for the cell lines and only kept those potential marker genes for further analysis that were in accordance with the transfer of the expression behavior from cell lines to tumors outlined above (overexpressed in radioresistant cell line vs. negative correlation between tumor expression and time until relapse, undexpressed in radioresistant cell line vs. positive correlation between tumor expression and time until relapse). This was fulfilled by 61 of 162 potential radioresistance driver genes from DU145 and for 14 of 27 from LNCaP (S5 Table). Finally, we analyzed each of these marker candidates for its potential to distinguish between early and late relapse of prostate cancer patients that received adjuvant radiation therapy. Therefore, we did a Kaplan-Meier analysis for each marker candidate where we tried to split the 32 irradiated TCGA prostate cancer patients into an early and late relapse group under consideration of the marker specific expression (R package ‘survival’ [74]). We determined an optimal gene expression cutoff for each marker for the separation into early and late relapse (disease free survival) by computing corresponding log-rank p-values with respect to the constraint that each group must contain at least eight patients. We selected all marker candidates with p-values less than 0.05 resulting in 10 markers from DU145 and 4 markers from LNCaP capable to distinguish between early and late relapse of irradiated prostate cancer patients for further analysis (S5 Table). The correlation between predicted and experimentally measured expression levels of these 14 candidate markers was significantly greater than zero (t-test: *P* < 0.019) and at the level of individual genes also significantly better than for random networks of same complexity derived by degree-preserving network permutations (paired t-test: *P* < 0.02). Corresponding estimated conservative false discovery rates were between 14% and 22% [44] and more liberal estimates between 3% and 5% [45] (S5 Table). Random selections of genes would have resulted on average on 0.90 genes for LNCaP and 2.25 genes for DU145 with log-rank p-values less than 0.05 (95% confidence interval [0.88, 0.91] for LNCaP and [2.22, 2.27] for DU145), which is significantly less than the number of driver candidates predicted by our network-based approach.

In addition, we used the ExaLT algorithm [46] to compute exact permutational log-rank p-values for each optimal candidate gene-specific split between early and late relapse patients. Our initially computed approximate log-rank p-values (R package ‘survival’) varied only marginally from the exact permutational p-values, except for *FOXL1* (increase in log-rank p-value from 0.014 to 0.076), supporting that our selection of driver gene candidates based on the small cohort of irradiated patients was robust (S4 Fig). We also used Cox regression [47, 48] (R package ‘survival’) to analyze if our driver candidates were still informative for disease-free survival in the presence of currently used prognostic factors (age, clinical T-Stage, Gleason score, psa). The grouping information about early or late relapse derived form each individual driver candidate was important to model disease-free survival and reached more significant p-values than the other covariates for 13 of 14 candidate genes (S7 Fig, *AKR1B10*: clinical T-stage was slightly more significant than the grouping information derived from *AKR1B10* expression).

Further, we used the determined optimal marker gene-specific expression cutoffs to analyze the 182 non-irradiated TCGA prostate cancer patients to determine those markers that were exclusively associated with relapse of irradiated patients but not with relapse of non-irradiated patients.

### Cell lines and culture conditions

Prostate cancer cell lines DU145, LNCaP and PC3 were purchased from the American Type Culture Collection (ATCC, Manassas, VA) and cultured according to the manufacturers recommendations in a humidified 37°C incubator supplemented with 5% CO_2_. DU145 and PC3 cells were maintained in Dulbecco’s Modified Eagle’s Medium (DMEM) (Sigma-Aldrich) and LNCaP cells in RPMI-1640 medium (Sigma-Aldrich) containing 10% fetal bovine serum (FBS, PAA Laboratories) and 1 mM L-glutamine (Sigma-Aldrich). The analyzed radioresistant cell lines of DU145 and LNCaP were established in [33] and further analyzed in [34]. In more detail, radioresistant cell sublines of DU145 and LNCaP had been generated by multiple fractions of 4 Gy X-ray irradiation until a total dose of more than 56 Gy was reached (Fig. 4a in [33]). Colony assays had been used to demonstrate the enhanced radioresistance of surviving cells (Fig. 4b in [33]). Corresponding age-matched non-irradiated radiosensitive parental cells were used as controls for radioresistant cell lines. All cell lines were genotyped using microsatellite polymorphism analysis and tested for mycoplasma directly before the experiments.

### Sphere formation assay

To evaluate the self-renewal potential, cells were grown as non-adherent multicellular cell aggregates (spheres). Cells were plated at a density of 1,000 cells/2 mL/well in 6-well ultra-low attachment plates (Corning) in MEBM medium (Lonza) supplemented with 4 μg/mL insulin (Sigma-Aldrich), B27 (Invitrogen), 20 ng/mL EGF (Peprotech), and 20 ng/mL FGF (Peprotech). Media containing supplements were refreshed once a week and spheres with a size > 100 μm were assayed after 14 days using Axiovert 25 microscope (Zeiss) or were automatically scanned using the Celigo S Imaging Cell Cytometer (Brooks).

### Knockdown of *VGF* by siRNA transfection

For knockdown of *VGF* expression, cells were transfected with RNAiMAX (Life Technologies GmbH) according to the manufacturer’s protocol. The siRNA target sequences were obtained from the Life Technologies website and corresponding RNA duplexes were synthesized by Eurofins. The sequences were VGF siRNA 1: sense GGAAGAAGCAGCUGAAGCUdCdT; antisense AGCUUCAGCUGCUUCUUCCdTdC and VGF siRNA 2: sense GGAGGAGCUGGAGAAUUACdAdT; antisense GUAAUUCUCCAGCUCCUCCdTdG for targeted knockdowns of *VGF*. Scrambled siRNA 1: sense UGCGCUAGGCCUCGGUUGCdTdT; antisense GCAACCGAGGCCUAGCGCAdTdT, scrambled siRNA 2: sense: AGGUAGUGUAAUCGCCUUGdTdT; antisense CAAGGCGAUUACACUACCUdTdT, and scrambled siRNA 3: sense GCAGCUAUAUGAAUGUUGUdTdT; antisense ACAACAUUCAUAUAGCUGCdTdT were used as negative control. In addition, knockdown efficiencies of VGF siRNA 1 and 2 were analyzed by RT-qPCR in comparison to scrambled siRNAs considering three biological replicates for DU145 and PC3 and two for LNCaP. Seven technical replicates were done for each biological replicate.

### Clonogenic cell survival assay

Cells were plated at a density of 500 cells/well in 6-well plates in complete medium and irradiated with doses of 2, 4 and 6 Gy of 200 kV X-rays (Yxlon Y.TU 320; dose rate 1.3 Gy/min at 20 mA) filtered with 0.5 mm Cu. The absorbed dose was measured using a Duplex dosimeter (PTW). After 10 days, the colonies were fixed with 10% formaldehyde (VWR) and stained with 0.05% crystal violet (Sigma-Aldrich). Colonies containing > 50 cells were counted using a stereo microscope (Zeiss). The plating efficiency (PE) was calculated as ratio between the number of colonies and the number of cells plated. The surviving fraction (SF) was calculated as ration between the PE of irradiated cells divided by PE of corresponding non-irradiated control cells. We also learned linear-quadratic (LQ) models to obtain a functional representation of the surviving fraction for each cell line using the R package ‘CFAssay’ [54] with standard settings (S11 Fig). We did not consider higher irradiation doses of 8 or 10 Gy in our experiments, because only few cells survived at 6 Gy especially for LNCaP and PC3.

## Supporting information

S1 Text Literature analysis and discussion of identified driver candidates. (PDF)

S1 Fig Technical flow chart. (PDF)

S2 Fig Heatmap representation of copy number alterations. (PDF)

S3 Fig Predictive power of network for cancer cell lines. (PDF)

S4 Fig Comparison of approximate and exact log-rank p-values. (PDF)

S5 Fig Marker gene-based separation of prostate cancer patients into early and late relapse groups. (PDF)

S6 Fig Copy number alteration levels of driver candidate genes. (PDF)

S7 Fig Cox regression results for modeling of disease-free survival. (PDF)

S8 Fig DU145, LNCaP and PC3: Monolayer vs. Spheres. (PDF)

S9 Fig Validation of VGF in prostate cancer cell line PC3. (PDF)

S10 Fig Western blots and RT-qPCR analysis. (PDF)

S11 Fig LQ model fits of clonogenic survival. (PDF)

S1 Table DNA copy number segmentation profiles of DU145 and LNCaP. (SEG)

S2 Table Gene copy number data of DU145 and LNCaP. (XLS)

S3 Table Gene expression data of DU145 and LNCaP. (XLS)

S4 Table Differentially expressed genes with directly underlying copy number alterations for DU145 and LNCaP. (XLS)

S5 Table Impacts of differentially expressed genes with directly underlying copy number alterations on known radioresistant marker genes. (XLS)

S6 Table Clinical information of irradiated and non-irradiated prostate cancer patients from TCGA. (XLS)

S7 Table Data of *VGF* validation experiments. (XLS)

S8 Table Connectivity table of prostate cancer-specific gene regulatory network. (TSV)
