# Supplementary material for: Network-based analysis of prostate cancer cell lines reveals novel marker gene candidates associated with radioresistance and patient relapse
Source: PLoS Comput Biol. 2019 Nov 4;15(11):e1007460. doi: 10.1371/journal.pcbi.1007460 (PMC6855562; doi:10.1371/journal.pcbi.1007460)
Supplement: S1 Text — (PDF) [file pcbi.1007460.s001.pdf]

Network-based analysis of prostate cancer cell lines reveals novel marker gene candidates associated with radioresistance and patient relapse

Michael Seifert, Claudia Peitzsch, Ielizaveta Gorodetska, Caroline Börner, Barbara Klink and Anna Dubrovska

Contact: michael.seifert@tu-dresden.de

S1 Text: Literature analysis and discussion of identified driver candidates

We revealed ten driver candidates from DU145 (*ADAMTS9*, *AKR1B10*, *CXXC5*, *FST*, *FOXL1*, *GRPR*, *ITGA2*, *SOX17*, *STARD4*, *VGF*) and four from LNCaP (*FHL5*, *LYPLAL1*, *PAK7*, *TDRD6*) that were able to distinguish irradiated prostate cancer patients into early and late relapse groups (Fig. 5 main manuscript). Here, we discuss these genes in the context of existing literature, except *VGF* that we already discussed in the main manuscript.

The metalloprotease ADAMTS9 (disintegrin-like and metalloproteinase with thrombospondin motifs) is a known tumor suppressor that is frequently epigenetically repressed in breast and colorectal tumors inhibiting cancer cell survival and migration via AKT and p53 signaling [1, 2]. Our study showed that low expression of *ASAMTS9* is associated with longer disease-free survival of prostate cancer patients treated with radiotherapy.

AKR1B10 (Aldo-keto reductase family 1, member B10) encodes an enzyme that is highly expressed in different cancers including breast, oral squamous cell carcinoma, and promotes tumor cell migration and invasion by activation of MAPK/ERK and FAK/Src/Rac1 signaling pathways [3, 4]. *AKR1B10* detoxifies different cytotoxic and carcinogenic carbonyl compounds and a recent study revealed that *AKR1B10* might efficiently prevent DNA damage induced by these compounds [5]. Importantly, *AKR1B10* is also involved in retinoic acid deprivation by reducing intracellular levels of all-trans- and 9-cis-retinaldehyde [6, 7]. In prostate cancer, retinoid acid was found to inhibit androgen receptor signaling and cell proliferation, to slow the tumor progression and to promote apoptosis [8]. High intracellular concentrations of retinoic acid also inhibit the aldehyde dehydrogenase (ALDH) activity and the translation of *ALDH2A1* [9], which is a marker of prostate cancer stem cells and a regulator of prostate cancer radioresistance [10, 11]. Therefore, *AKR1B10* might contribute to cancer cell survival and tumor relapse after radiotherapy by deprivation of retinoic acid and detoxification of carbonyl compounds. Consistent to this hypothesis, our study showed that low expression of *AKR1B10* correlates with longer disease-free survival of prostate cancer patients treated with radiotherapy.

CXXC5 encodes a transcriptional activator containing a CXXC-type zinc finger domain [12]. The expression of *CXXC5* is upregulated in acute myeloid leukemia, papillary thyroid carcinoma, metastatic melanoma and breast cancer and has been associated with adverse prognosis in breast tumors and acute myeloid leukemia [13, 14]. Recent studies of mRNA and protein expression in 65 needle-biopsy specimens from patients with localized prostate cancer showed that expression of *CXXC5* is significantly higher in tumors and precancerous prostate lesions such as high grade prostate intra-epithelial neoplasia (HGPIN) and proliferative inflammatory atrophy (PIA) compared to benign prostate tissue [15]. This suggests a role of *CXXC5* in prostate tumor initiation, although the mechanisms how *CXXC5* contributes to cancer development remain unclear. A study on breast cancer cells showed that upon DNA damage, *CXXC5* plays a role in activation of ataxia–telangiectasia-mutated (ATM)-p53 signaling following growth

inhibition and activation of DNA double strand break repair, but a knockdown of *CXXC5* did not affect the recruitment of ATM to the sites of double strand breaks suggesting that the role of *CXXC5* in the DNA damage signaling pathway is rather dispensable [16]. Importantly, a growing body of evidence suggests that *CXXC5* is an inhibitor of WNT/beta-catenin signaling in different tissue types including osteoblasts, leukemia cells and neural stem cells [17–20]. Our previous studies showed that the activation of WNT/beta-catenin signaling regulates radioresistance in prostate cancer progenitor cells and that the inhibition of the WNT pathway results in tumor cell radiosensitization [10, 21]. In agreement with this, our current study showed that high expression of the WNT inhibitor *CXXC5* is associated with longer disease-free survival of prostate cancer patients treated with radiotherapy.

GRPR encoding a gastrin-releasing peptide receptor is overexpressed in many types of cancers and is an attractive target for diagnostic imaging and radionuclide therapy [22]. The data on *GPRP* as prognostic biomarker in prostate cancer is controversial. A study by Beer *et al.* with 530 prostate cancer patients found that low expression of *GRPR* correlates with a worse Gleason score, high preoperative PSA levels and larger tumor sizes [23]. In contrast, a study by Nagasaki *et al.* with 51 human prostate cancer cases showed opposite results [24]. Further, some studies found that high expression of *GRPR* induces EMT in prostate tumor cells and that this is predictive for bone metastasis [25, 26]. Our study showed that expression of *GRPR* is lower in both considered radioresistant cell lines (DU145: andorgen independent, LNCaP: androgen dependent) and that low expression of *GRPR* is associated with shorter disease-free survival of prostate cancer patients treated with radiotherapy.

FOXL1 encodes a transcription factor involved in cell proliferation and differentiation that has been reported as tumor suppressor in pancreatic cancer [27]. Better clinical outcomes of pancreatic ductal adenocarcinoma were correlated with increased *FOXL1* expression, whereas reduced expression of *FOXL1* was correlated with metastasis and advanced stages of pancreatic cancer [27]. Mechanistic analyses showed that overexpression of *FOXL1* induced apoptosis and inhibited proliferation and invasion, whereas silencing of *FOXL1* inhibited apoptosis and enhanced cell growth and invasion of pancreatic cancer cells [27]. Further, *FOXL1* can activate WNT/beta-catenin signaling in the gut by increasing extracellular proteoglycans [28]. This *FOXL1*-triggered WNT signaling is required to maintain an active intestinal stem cell niche [29]. Thus, *FOXL1* might counteract an inhibition of WNT signaling by the marker gene *CXXC5*. Our study showed that high expression of *FOXL1* is correlated with longer disease-free survival of prostate cancer patients treated with radiotherapy.

FST encodes a gonadal protein (follistatin) that was first identified in ovarian follicular fluid and found to inhibit the release of the follicle-stimulating hormone [30]. More recent studies also suggest that *FST* is further involved in delayed apoptosis under glucose-deprived conditions [31] and metastases formation in a breast cancer mouse model [32]. Increased expression of *FST* in breast cancer has recently been associated with reduced invasion and better survival [33]. Similarly, our study showed that high expression of *FST* is associated with longer disease-free survival of prostate cancer patients treated with radiotherapy.

SOX17 encodes a transcription factor that is involved in embryonic development and cell fate determination [30]. Low *SOX17* expression has been associated with tumor progression and poor prognosis of breast cancer [34] and with poor prognosis of esophageal cancer [35]. *SOX17* has been shown to represent a key regulator of tumor angiogenesis and tumor progression in mice [36]. Tumors with higher *SOX17* expression might also be more sensitive to cisplatin treatment [37]. Further, *SOX17* has recently been shown to restrain proliferation and tumor formation by inhibiting WNT/beta-catenin signaling in cervical cancer [38]. Thus, like the marker gene *CXXC5*, *SOX17* might contribute to an inhibition of WNT signaling observed for radiosensitive prostate cancer [10, 21]. In accordance, we found that high expression of *SOX17* is associated with longer disease-free survival of prostate cancer patients treated with radiotherapy.

PAK7 encodes a serine/threonine protein kinase involved in different signaling pathways including cy-

toskeleton regulation, cell migration, proliferation and cell survival [30]. A knockdown of *PAK7* has been found to inhibit cell proliferation in gastric cancer [39]. In contrast, we found that high expression of *PAK7* is associated with longer disease-free survival of prostate cancer patients treated with radiotherapy.

ITGA2 encodes the alpha subunit of a transmembrane receptor for collagens, laminin, fibronectin, and E-caderin involved in the regulation of the extracellular matrix [30]. Antibody-based blocking of *ITGA2* has been reported to inhibit cell migration and to induce apoptosis in gastric cancer cells [40]. In contrast, the inhibition of *ITGA2* expression has been found to promote cell migration in breast cancer [41]. We found that high expression of *ITGA2* is associated with longer disease-free survival of prostate cancer patients treated with radiotherapy.

So far, no cancer-driving roles have been reported for the marker genes *STARD4*, *FHL5*, *TDRD6*, and *LYPLAL1*. *STARD4* is involved in the intracellular transport of sterols between the endocytic recycling compartment and the plasma membrane [42]. *FHL5* is potentially involved in spermatogenesis and its closely related protein family member *FHL1* has recently been shown to play a role in radiation resistance [43]. Also *TDRD6* is involved in spermatogenesis. *LYPLAL1* has similarity to phospholipases and hydrolyzes short chain substrates, but does not show phospholipase or triacylglycerol lipase activity [44]. We found that high expression levels of these genes were associated with longer disease-free survival of prostate cancer patients treated with radiotherapy.

References

[1] B. Shao, Y. Feng, H. Zhang, F. Yu, Q. Li, C. Tan, et al. The 3p14.2 tumour suppressor ADAMTS9 is inactivated by promoter CpG methylation and inhibits tumour cell growth in breast cancer. *J Cell Mol Med*, 22(2):1257–71, 2018.

[2] L. Chen, J. Tang, Y. Feng, S. Li, Q. Xiang, X. He, et al. ADAMTS9 is Silenced by Epigenetic Disruption in Colorectal Cancer and Inhibits Cell Growth and Metastasis by Regulating Akt/p53 Signaling. *Cell Physiol Biochem*, 44(4):1370–80, 2017.

[3] C. Huang, S. Verhulst, Y. Shen, Y. Bu, Y. Cao, Y. He, et al. AKR1B10 promotes breast cancer metastasis through integrin alpha5/delta-catenin mediated FAK/Src/Rac1 signaling pathway. *Oncotarget*, 7(28):43779–91, 2016.

[4] J. Li, Y. Guo, L. Duan, X. Hu, X. Zhang, J. Hu, et al. AKR1B10 promotes breast cancer cell migration and invasion via activation of ERK signaling. *Oncotarget*, 8:33694–703, 2017.

[5] X. Zu, R. Yan, J. Pan, L. Zhong, Y. Cao, J. Ma, et al. Aldo-keto reductase 1B10 protects human colon cells from DNA damage induced by electrophilic carbonyl compounds. *Mol Carcinog*, 56(1):118–29, 2017.

[6] F. X. Ruiz, S. Porte, X. Parés, and J. Farrés. Biological role of aldo-keto reductases in retinoic Acid biosynthesis and signaling. *Front Pharmacol*, 3:58, 2012.

[7] F. X. Ruiz, O. Gallego, A. Ardèvol, A. Moro, M. Domínguez, and S. Alvarez. Aldo-keto reductases from the AKR1B subfamily: retinoid specificity and control of cellular retinoic acid levels. *Chem Biol Interact*, 178(1-3):171–7, 2009.

[8] M. C. Chen, S. L. Hsu, H. Lin, and T. Y. Yang. Retinoic acid and cancer treatment. *Biomedicine (Taipei)*, 4:22, 2014.

[9] J. S. Moreb, A. Gabr, G. R. Vartikar, S. Gowda, J. R. Zucali, and D. Mohuczy. Retinoic acid down-regulates aldehyde dehydrogenase and increases cytotoxicity of 4-hydroperoxycyclophosphamide and acetaldehyde. *J Pharmacol Exp Ther*, 312(1):339–45, 2005.

[10] M. Cojoc, C. Peitzsch, I. Kurth, F. Trautmann, L. A. Kunz-Schughart, G. D. Telegeev, et al. Aldehyde Dehydrogenase Is Regulated by beta-Catenin/TCF and Promotes Radioresistance in Prostate Cancer Progenitor Cells. *Cancer Res*, 75(7):1482–94, 2015.

[11] P. E. Burger, R. Gupta, X. Xiong, C. S. Ontiveros, S. N. Salm, D. Moscatelli, et al. High aldehyde dehydrogenase activity: a novel functional marker of murine prostate stem/progenitor cells. *Stem Cells*, 27(9):2220–8, 2009.

[12] F. Pendino, E. Nguyen, I. Jonassen, B. Dysvik, A. Azouz, M. Lanotte, et al. Functional involvement of RINF, retinoid-inducible nuclear factor (CXXC5), in normal and tumoral human myelopoiesis. *Blood*, 113(14):3172–81, 2009.

[13] S. Knappskog, L. M. Myklebust, C. Busch, T. Aloysius, J. E. Varhaug, P. E. Lonning, et al. RINF (CXXC5) is overexpressed in solid tumors and is an unfavorable prognostic factor in breast cancer. *Ann Oncol*, 22(10):2208–15, 2011.

[14] O. Bruserud, H. Reikvam, H. Fredly, J. Skavland, K. M. Hagen, T. T. van Hoang, et al. Expression of the potential therapeutic target CXXC5 in primary acute myeloid leukemia cells - high expression is associated with adverse prognosis as well as altered intracellular signaling and transcriptional regulation. *Oncotarget*, 6(5):2794–811, 2015.

[15] I. Benedetti, A. M. De Marzo, J. Geliebter, and N. Reyes. CXXC5 expression in prostate cancer: implications for cancer progression. *Int J Exp Pathol*, 98(4):234–43, 2017.

[16] M. Zhang, R. Wang, Y. Wang, F. Diao, F. Lu, D. Gao, et al. The CXXC finger 5 protein is required for DNA damage-induced p53 activation. *Sci China C Life Sci*, 52(6):528–38, 2009.

[17] A. Kuhnl, P. J. Valk, M. A. Sanders, A. Ivey, R. K. Hills, K. I. Mills, et al. Downregulation of the Wnt inhibitor CXXC5 predicts a better prognosis in acute myeloid leukemia. *Blood*, 125(19):2985–94, 2015.

[18] T. Andersson, E. Sodersten, J. K. Duckworth, A. Cascante, N. Fritz, P. Sacchetti, et al. CXXC5 is a novel BMP4-regulated modulator of Wnt signaling in neural stem cells. *J Biol Chem*, 284(6):3672–81, 2009.

[19] D. Kim and L. A. Garza. The Negative Regulator CXXC5: Making WNT Look a Little Less Dishevelled. *J Invest Dermatol*, 137(11):2248–50, 2017.

[20] H. Y. Kim, J. Y. Yoon, J. H. Yun, K. W. Cho, S. H. Lee, Y. M. Rhee, et al. CXXC5 is a negative-feedback regulator of the Wnt/beta-catenin pathway involved in osteoblast differentiation. *Cell Death Differ*, 22(6):912–20, 2015.

[21] C. Peitzsch, M. Cojoc, L. Hein, I. Kurth, Mabert K., F. Trautmann, et al. An epigenetic reprogramming strategy to resensitize radioresistant prostate cancer cells. *Cancer Res*, 76:2637–51, 2016.

[22] L. Baratto, H. Jadvar, and A. Iagaru. Prostate Cancer Theranostics Targeting Gastrin-Releasing Peptide Receptors. *Mol Imaging Biol*, 20(4):501–9, 2018.

[23] M. Beer, M. Montani, J. Gerhardt, P. J. Wild, T. F. Hany, T. Hermanns, et al. Profiling gastrin-releasing peptide receptor in prostate tissues: clinical implications and molecular correlates. *Prostate*, 72(3):318–25, 2012.

[24] S. Nagasaki, Y. Nakamura, T. Maekawa, J. Akahira, Y. Miki, T. Suzuki, et al. Immunohistochemical analysis of gastrin-releasing peptide receptor (GRPR) and possible regulation by estrogen receptor betacx in human prostate carcinoma. *Neoplasma*, 59(2):224–32, 2012.

[25] S. M. Elshafae, B. B. Hassan, W. P. Supsavhad, W. ans Dirksen, R. Y. Camiener, H. Ding, et al. Gastrin-releasing peptide receptor (GRPr) promotes EMT, growth, and invasion in canine prostate cancer. *Prostate*, 76(9):796–809, 2016.

[26] H. J. Ananias, M. C. van den Heuvel, W. Helfrich, and I. J. de Jong. Expression of the gastrin-releasing peptide receptor, the prostate stem cell antigen and the prostate-specific membrane antigen in lymph node and bone metastases of prostate cancer. *Prostate*, 69(10):1101–8, 2009.

[27] G. Zhang, P. He, J. Gaedcke, B. M. Ghadimi, T. Ried, H. G. Yfantis, et al. FOXL1, a novel candidate tumor suppressor, inhibits tumor aggressiveness and predicts outcome in human pancreatic cancer. *Cancer Res*, 73(17):5416–25, 2013.

[28] N. Perreault, J. P. Katz, S. D. Sackett, and K. H. Kaestner. Foxl1 controls the Wnt/beta-catenin pathway by modulating the expression of proteoglycans in the gut. *J Biol Chem*, 276(46):43328–33, 2001.

[29] R. Aoki, M. Shoshkes-Carmel, N. Gao, S. Shin, C. L. May, M. L. Golson, et al. Foxl1-expressing mesenchymal cells constitute the intestinal stem cell niche. *Cell Mol Gastroenterol Hepatol*, 2(2):175–88, 2016.

[30] M. Safran, I. Dalah, J. Alexander, N. Rosen, T. Iny Stein, M. Shmoish, et al. GeneCards version 3: the human gene integrator. *Database*, 2010:baq020, 2010.

[31] X. Gao, S. Wei, K. Lai, J. Sheng, J. Su, J. Zhu, et al. Nucleolar follistatin promotes cancer cell survival under glucose-deprived conditions through inhibiting cellular rRNA synthesis. *J Biol Chem*, 285(47):36857–64, 2010.

[32] D. D. Seachrist, S. T. Sizemore, E. Johnson, F. W. Abdul-Karim, K. L. Weber Bonk, and R. A. Keri. Follistatin is a metastasis suppressor in a mouse model of HER2-positive breast cancer. *Breast Cancer Res*, 19(1):66, 2017.

[33] C. Zabkiewicz, J. Resaul, R. Hargest, W. G. Jiang, and L. Ye. Increased Expression of Follistatin in Breast Cancer Reduces Invasiveness and Clinically Correlates with Better Survival. *Cancer Genomics Proteomics*, 14(4):241–251, 2017.

[34] D. Y. Fu, H. S. Tan, J. L. Wei, C. R. Zhu, J. X. Jiang, Y. X. Zhu, et al. Decreased expression of SOX17 is associated with tumor progression and poor prognosis in breast cancer. *Tumour Biol*, 36(10):8025–34, 2015.

[35] I. Y. Kuo, C. C. Wu, J. M. Chang, Y. L. Huang, C. H. Lin, et al. Low SOX17 expression is a prognostic factor and drives transcriptional dysregulation and esophageal cancer progression. *Int J Cancer*, 135(3):563–73, 2014.

[36] H. Yang, S. Lee, S. Lee, K. Kim, Y. Yang, J. H. Kim, et al. Sox17 promotes tumor angiogenesis and destabilizes tumor vessels in mice. *J Clin Invest*, 123(1):418–31, 2013.

[37] Y. Zhang, F. Jiang, W. Bao, H. Zhang, X. He, H. Wang, et al. SOX17 increases the cisplatin sensitivity of an endometrial cancer cell line. *Cancer Cell Int*, 16:29, 2016.

[38] G. Zhang, P. He, J. Gaedcke, B. M. Ghadimi, T. Ried, H. G. Yfantis, et al. SOX17 restrains proliferation and tumor formation by down-regulating activity of the wnt/β-catenin signaling pathway via trans-suppressing β-catenin in cervical cancer. *Cell Death Dis*, 9(7):741, 2018.

[39] J. Gu, K. Li, M. Li, X. Wu, L. Zhang, Q. Ding, et al. A role for p21-activated kinase 7 in the development of gastric cancer. *FEBS J*, 280(1):46–55, 2013.

[40] Y. C. Chuang, H. Y. Wu, Y. L. Lin, S. C. Tzou, C. H. Chuang, T. Y. Jian, et al. Blockade of ITGA2 Induces Apoptosis and Inhibits Cell Migration in Gastric Cancer. *Biol Proced Online*, 20:10, 2018.

[41] W. Ding, X. L. Fan, X. Xu, J. Z. Huang, S. H. Xu, Q. Geng, et al. Epigenetic silencing of ITGA2 by MiR-373 promotes cell migration in breast cancer. *PloS One*, 10(8):e0135128, 2015.

[42] D. B. Iaea, S. Mao, F. W. Lund, and F. R. Maxfield. Role of STARD4 in sterol transport between the endocytic recycling compartment and the plasma membrane. *Mol Biol Cell*, 28(8):1111–22, 2017.

[43] X. Xu, Z. Fan, C. Liang, L. Li, L. Wang, Y. Liang, et al. A signature motif in LIM proteins mediates binding to checkpoint proteins and increases tumour radiosensitivity. *Nat Commun*, 8:14059, 2017.

[44] M. Bürger, T. J. Zimmermann, Y. Kondoh, P. Stege, N. Watanabe, H. Osada, et al. Crystal structure of the predicted phospholipase LYPLAL1 reveals unexpected functional plasticity despite close relationship to acyl protein thioesterases. *J Lipid Res*, 53(1):43–50, 2012.
