# Supplementary material for: Network-based analysis of prostate cancer cell lines reveals novel marker gene candidates associated with radioresistance and patient relapse
Source: PLoS Comput Biol. 2019 Nov 4;15(11):e1007460. doi: 10.1371/journal.pcbi.1007460 (PMC6855562; doi:10.1371/journal.pcbi.1007460)
Supplement: S1 Fig — (PDF) [file pcbi.1007460.s002.pdf]

S1 Figure: Technical flow chart.

TCGA prostate cancer patients

- gene copy number and expression profiles
- 541 patients
- 14,780 genes

Inference of prostate cancer-specific network

- R package regNet
- computing time: 670 hours
- filters: links with p-values ≈ 0, local gene cutoff 50
- validation: gene expression prediction for 768 CCLE cancer cell lines, comparison to random networks

Network-based impact quantification for DU145/LNCaP

- R package regNet
- network propagation: absolute impacts
- input: DU145 and LNCaP gene copy number and expression profiles for radioresistant vs. radiosensitive, gene-specific predicitive power from CCLE
- output: cell line-specific genome-wide impact matrix
- analysis of impacts of differentially expressed genes with directly underlying gene copy number alterations on radioresistance markers
    - 292 genes for radioresistant vs. radiosensitive DU145
    - 40 genes for radioresistant vs. radiosensitive LNCaP
- comparison to impacts under random networks
- 162 driver candidates for DU145 and 27 for LNCaP (q-value < 0.01)

Transfer of driver candidates to prostate cancer patients

- 214 of 541 TCGA prostate cancer patients with sufficient meta-information
- 32 of 214 received radiation and 182 of 214 did not
- filter: driver candidates with consistent expression between DU145/LNCaP and tumor samples of irradiated patients with relapse (12 of 32)
    - 61 of 162 driver candidates from DU145 and 14 of 27 from LNCaP
- Kaplan-Meier-Analysis: Distinguish between early and late relapse by gene expression-based optimal grouping of 32 irradiated patients, where each group must contain at least 8 patients
    - log-rank p-value < 0.05
    - 10 of 61 DU145 and 4 of 27 LNCaP candidates
    - FDR estimates: 14-22% (conservative), 3-5% (liberal)

Validation of VGF by in vitro radiobiological assays

- VGF expression analysis in independent cell lines
- VGF knockdown in combination with clonogenic cell survival assays and DNA double strand break repair efficiency

S1 Figure: Technical flow chart that outlines details of our developed computational data analysis pipeline for the identification of potential radioresistance driver genes. See Fig. 1 in main manuscript for a high-level overview.
