# Supplementary material for: Network-based analysis of prostate cancer cell lines reveals novel marker gene candidates associated with radioresistance and patient relapse
Source: PLoS Comput Biol. 2019 Nov 4;15(11):e1007460. doi: 10.1371/journal.pcbi.1007460 (PMC6855562; doi:10.1371/journal.pcbi.1007460)
Supplement: S2 Fig — (PDF) [file pcbi.1007460.s003.pdf]

S2 Figure: Heatmap representation of copy number alterations.

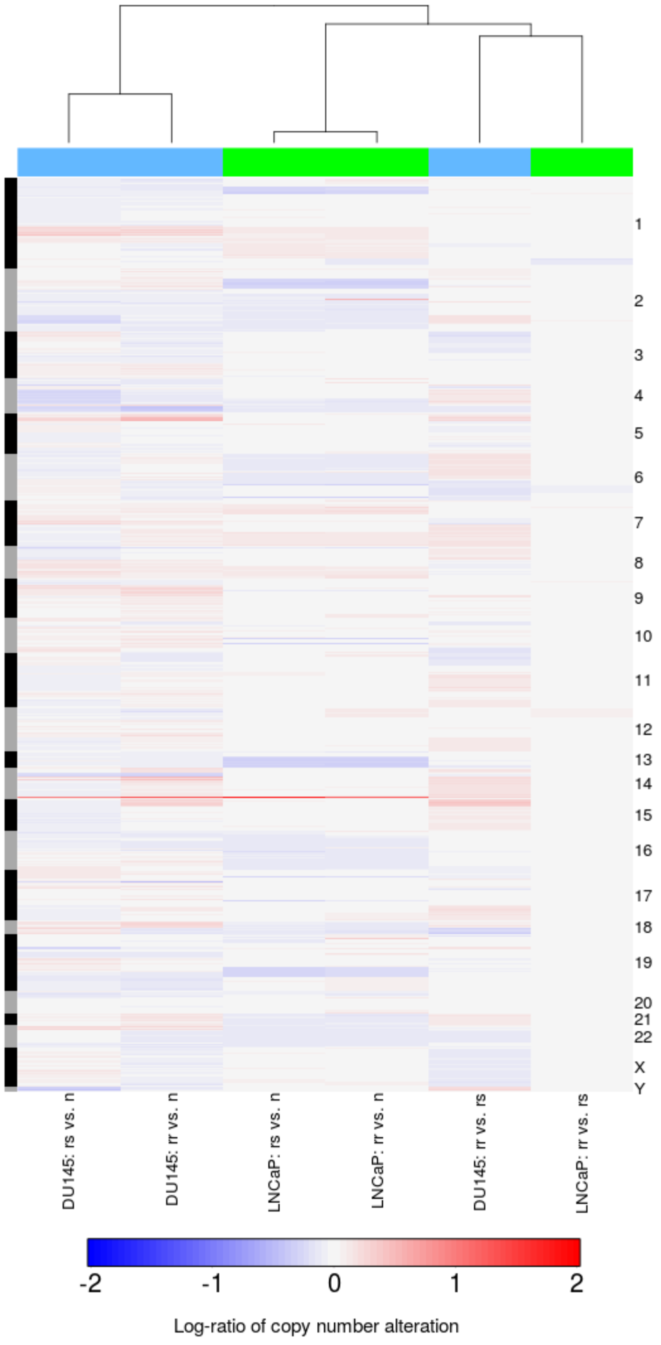

S2 Figure: Heatmap representing genome-wide gene copy number alterations of DU145 and LNCaP cell lines. The columns represent the log-ratio profiles of DU145 and LNCaP comparing either radiosensitive (rs) to normal (n), radioresistant (rr) to normal, or radioresistant to radiosensitive. The normal sample represents an Agilent Euro Male probe that was used as reference in the aCGH experiments. Reduced copy numbers are shown in blue shades, increased copy numbers are shown in red shades, and unchanged copy numbers are shown in light grey for each specific comparison along the chromosomes from 1 to Y. Chromosomes are also highlighted by alternating black and grey bars on the left side of the heatmap. Ward clustering was used to group the log-ratio profiles according to their similarities. Similarities and differences of copy number alterations are clearly visible. Considering the comparison to the normal reference, radioresistant and radiosensitive samples of each cell line cluster together (rr vs. n, rs vs. n). The direct comparison of radioresistant and radiosensitive samples of each cell line forms a separate cluster where DU145 shows much more copy number alterations than LNCaP (rr vs. rs).
