# Supplementary material for: Network-based analysis of prostate cancer cell lines reveals novel marker gene candidates associated with radioresistance and patient relapse
Source: PLoS Comput Biol. 2019 Nov 4;15(11):e1007460. doi: 10.1371/journal.pcbi.1007460 (PMC6855562; doi:10.1371/journal.pcbi.1007460)
Supplement: S3 Fig — (PDF) [file pcbi.1007460.s004.pdf]

S3 Figure: Predictive power of network for cancer cell lines.

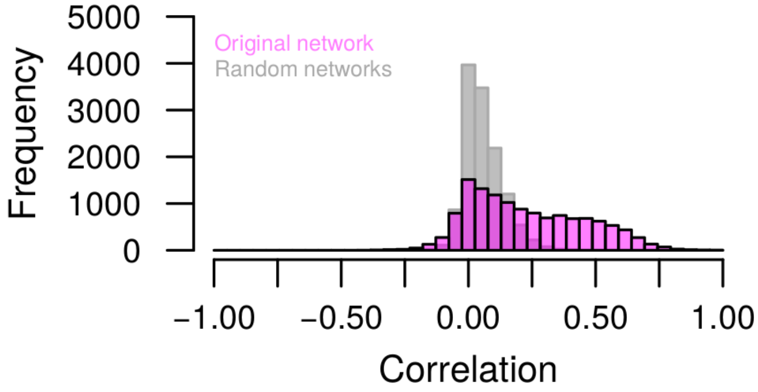

S3 Figure: The prostate cancer specific network learned based on gene expression and gene copy number data of 541 prostate cancer patients from The Cancer Genome Atlas (TCGA) was used to predict the expression levels of individual genes for 768 cancer cell lines from the Cancer Cell Line Encyclopedia (CCLE) (Barretina et al. (2012), Seifert et al. (2016)). Pearson correlations between predicted and originally measured expression levels were determined for each of the 14,780 genes considering the original network (lilac) and ten random networks (gray) of same complexity (degree-preserving network permutations). The correlation distribution of the original network is significantly shifted into the positive range ($P \approx 0$, Wilcoxon test). The original network is also significantly better than the random networks at the population and the single gene level ($P \approx 0$, unpaired and paired Wilcoxon tests). The reached predictive power for a gene is the better the more positive the reached correlation is. These gene-specific correlations were included in the network propagation computations to give more weight to genes that were better predictable. See Seifert et al. (2016) and Seifert et al. (2018) for computational details of the network propagation algorithm.
