# Supplementary material for: Network-based analysis of prostate cancer cell lines reveals novel marker gene candidates associated with radioresistance and patient relapse
Source: PLoS Comput Biol. 2019 Nov 4;15(11):e1007460. doi: 10.1371/journal.pcbi.1007460 (PMC6855562; doi:10.1371/journal.pcbi.1007460)
Supplement: S4 Fig — (PDF) [file pcbi.1007460.s005.pdf]

## S4 Figure: Comparison of approximate and exact log-rank p-values.

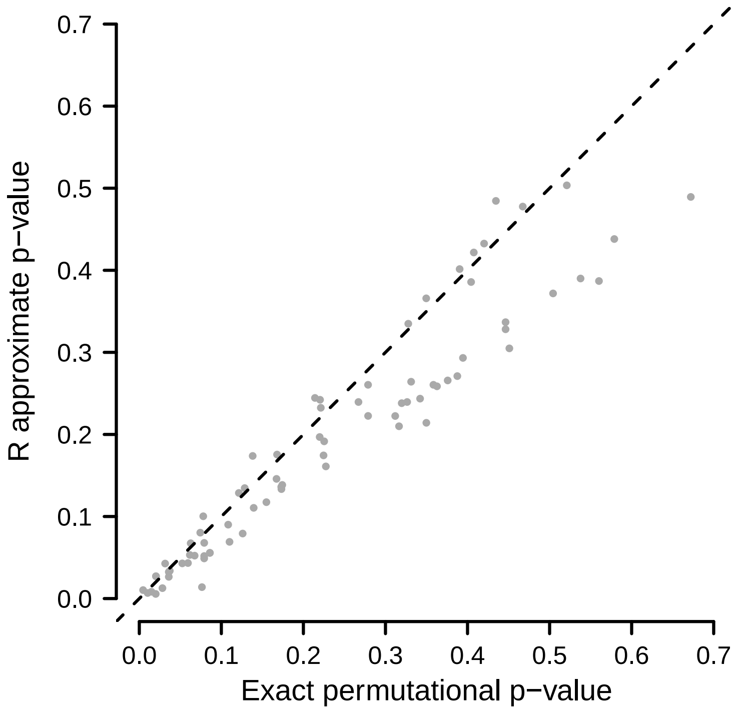


**S4 Figure:** Comparison of approximate and exact log-rank p-values of driver gene candidates of DU145 and LNCaP. A separation of the 32 irradiated prostate cancer patients from TCGA into an early and late relapse group was done based on the optimal expression cutoff of each marker gene as described in the main manuscript. Approximate log-rank p-values quantify for each marker gene the difference in disease-free survival and were computed by the R function survdiff. Because approximate p-values can be biased especially for the analysis of a small cohort with two different-sized subgroups, we additionally computed corresponding exact permutational p-values using the ExaLT method for all driver candidate genes (Vandin et al. (2015)). The scatter plot visualizes deviations between the approximate and the exact log-rank p-value of each marker gene (grey dots). Mainly an overestimation of the significance by approximate log-rank p-values is observed (grey dots below main diagonal), but this only slightly affected our selected candidate genes with small p-values, whereas this effect was much more pronounced for larger insignificant p-values. Considering our 14 candidate genes with approximate log-rank p-values less than 0.05, only *FOXL1* showed a large increase of the log-rank p-value from 0.014 (approximate) to 0.076 (exact), whereas the other candidates showed marginal deviations in both directions leading to slightly improved or slightly worse log-rank p-values.
