# Supplementary material for: Network-based analysis of prostate cancer cell lines reveals novel marker gene candidates associated with radioresistance and patient relapse
Source: PLoS Comput Biol. 2019 Nov 4;15(11):e1007460. doi: 10.1371/journal.pcbi.1007460 (PMC6855562; doi:10.1371/journal.pcbi.1007460)
Supplement: S5 Fig — (PDF) [file pcbi.1007460.s006.pdf]

S5 Figure: Marker gene-based separation of prostate cancer patients into early and late relapse groups.

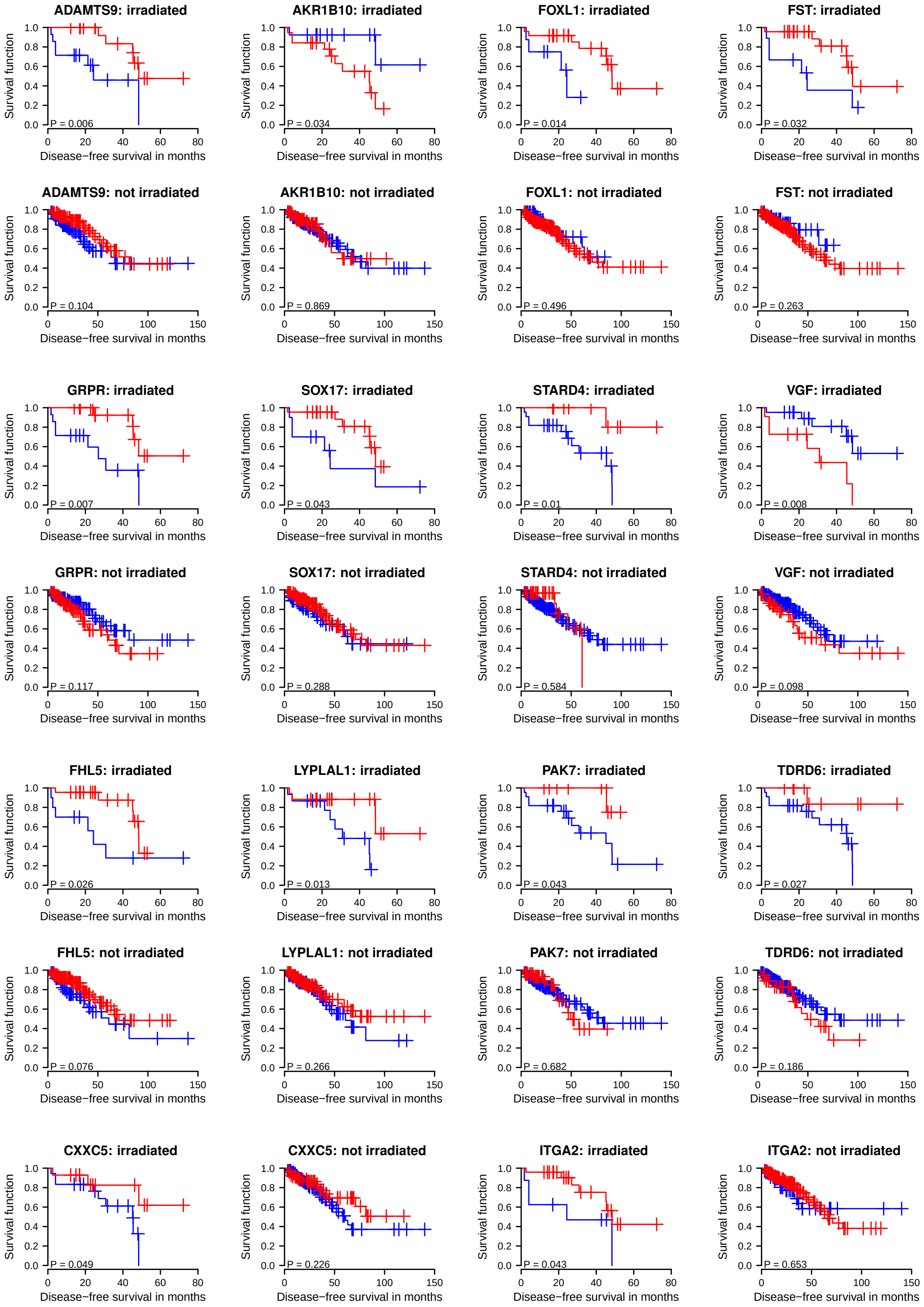

S5 Figure: Potential radioresistance driver genes revealed form DU145 and LNCaP were analyzed for their expression behavior in 32 irradiated and 182 non-irradiated prostate cancer patients available from TCGA. Expression levels of each marker gene across the 32 irradiated patients were used to determine a marker gene-specific optimal cutoff for disease-free survival risk curves separating patients with low (blue curve) and high (red curve) marker gene expression with respect to the constraint that at least 8 patients must be assigned to each curve. These optimal cutoffs were further used to analyze the separation behavior for the non-irradiated patients. Log-rank test p-values indicate that these selected marker genes enable a separation of irradiated prostate cancer patients into early and late relapse groups, whereas this separation potential is lost for the majority of markers considering non-irradiated patients, except *VGF* and *FHL5*.
