# Supplementary material for: Network-based analysis of prostate cancer cell lines reveals novel marker gene candidates associated with radioresistance and patient relapse
Source: PLoS Comput Biol. 2019 Nov 4;15(11):e1007460. doi: 10.1371/journal.pcbi.1007460 (PMC6855562; doi:10.1371/journal.pcbi.1007460)
Supplement: S6 Fig — (PDF) [file pcbi.1007460.s007.pdf]

S6 Figure: Copy number alteration levels of driver candidate genes.

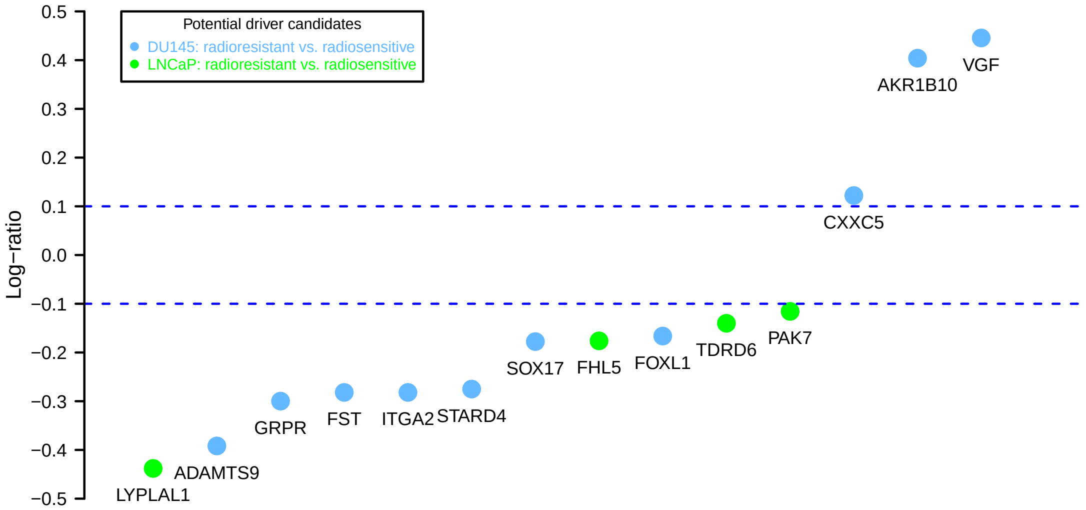

S6 Figure: Measured copy number alteration levels of predicted driver candidate genes with strong impact on the expression of radioresistance marker genes. Log_2-ratios of copy number alteration levels comparing radioresistant to radiosensitive cell lines of DU145 or LNCaP are shown on the y-axis. The genes are sorted according to increasing log-ratios and visualized by dots that are colored according to the cell line in which a candidate gene has been predicted. The two blue dashed horizontal lines mark the log-ratio cutoff that was used to select these genes from our aCGH data.
