# Supplementary material for: Network-based analysis of prostate cancer cell lines reveals novel marker gene candidates associated with radioresistance and patient relapse
Source: PLoS Comput Biol. 2019 Nov 4;15(11):e1007460. doi: 10.1371/journal.pcbi.1007460 (PMC6855562; doi:10.1371/journal.pcbi.1007460)
Supplement: S7 Fig — (PDF) [file pcbi.1007460.s008.pdf]

S7 Figure: Cox regression results for modeling of disease-free survival.

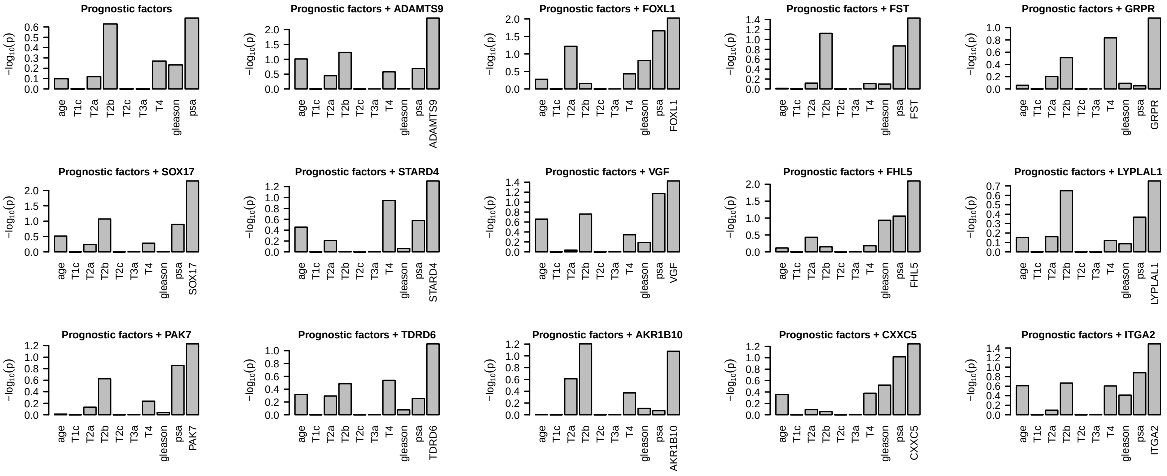

S7 Figure: Contributions of driver gene candidates to the modeling of disease-free survival in the presence of other prognostic factors (age, clinical T-stage (T1c, T2a, T2b, T2c, T3a, T4), Gleason score, psa). Clinical information of all 32 irradiated prostate cancer patients from TCGA were considered to model the disease-free survival time by Cox regression (Cox (1972), Andersen et al. (1982)) to determine the contribution of each driver gene candidate in relation to the other prognostic factors. The barplots show the $-log_{10}(p)$ of each p-value p obtained for the individual covariates in the corresponding regression model. The subfigure 'Prognostic factors' shows that none of the currently used clinical factors is well-suited to model disease-free survival (top-left). The other subfigures show that each driver candidate-based separation into an early and late relapse group provides important information to model disease-free survival. Only the contribution of one candidate gene (AKR1B10) is slightly less significant than the clinical T-stage.
