# Supplementary material for: Network-based analysis of prostate cancer cell lines reveals novel marker gene candidates associated with radioresistance and patient relapse
Source: PLoS Comput Biol. 2019 Nov 4;15(11):e1007460. doi: 10.1371/journal.pcbi.1007460 (PMC6855562; doi:10.1371/journal.pcbi.1007460)
Supplement: S8 Fig — (PDF) [file pcbi.1007460.s009.pdf]

S8 Figure: DU145, LNCaP and PC3: Monolayer vs. Spheres.

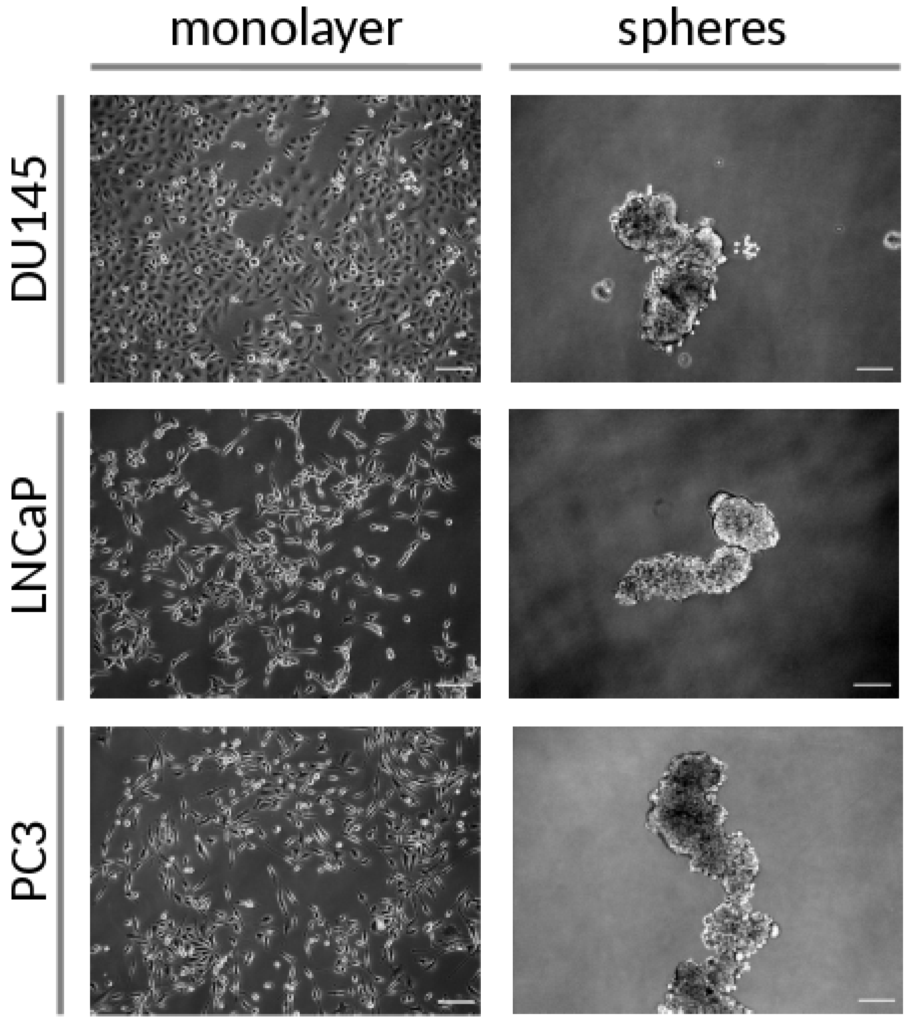

S8 Figure: Representative examples of microscope images of the cell lines DU145, LNCaP and PC3 grown under monolayer and sphere forming conditions. Scale bar size 100 µm. Parental DU145 and LNCaP cell lines were considered for this analysis.
