# Supplementary material for: Network-based analysis of prostate cancer cell lines reveals novel marker gene candidates associated with radioresistance and patient relapse
Source: PLoS Comput Biol. 2019 Nov 4;15(11):e1007460. doi: 10.1371/journal.pcbi.1007460 (PMC6855562; doi:10.1371/journal.pcbi.1007460)
Supplement: S9 Fig — (PDF) [file pcbi.1007460.s010.pdf]

S9 Figure: Validation of *VGF* in prostate cancer cell line PC3.

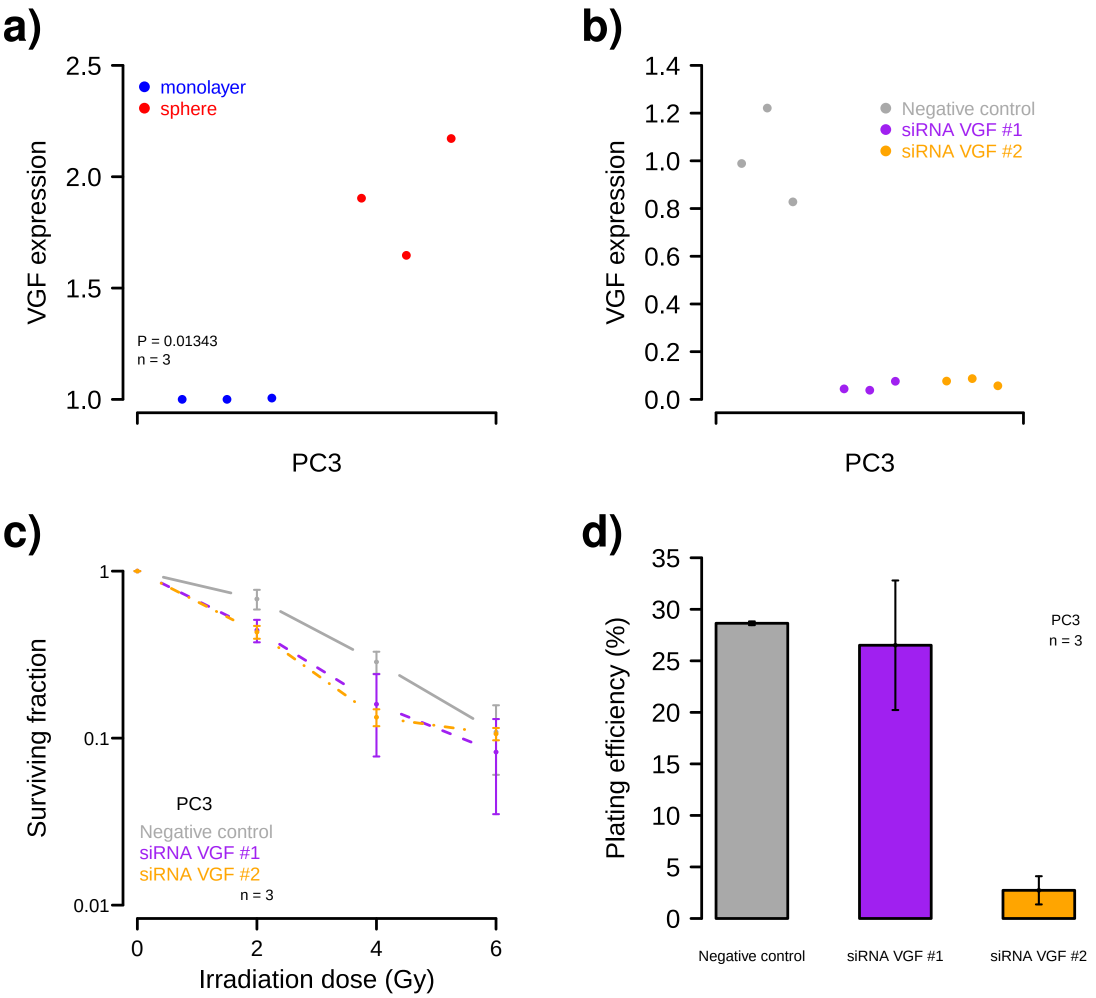

S9 Figure: Additional validation of *VGF* in the prostate cancer cell line PC3. (a) *VGF* expression in sphere cultures relative to monolayer cultures. Measurements were obtained by RT-qPCR for three biological replicates (S7 Table). See S8 Figure for microscope images of PC3 grown under monolayer and sphere conditions. (b) Efficiency of *VGF* knockdowns by siRNA VGF #1 and siRNA VGF #2 relative to negative control (scrambled siRNAs). Measurements were obtained by RT-qPCR for three biological replicates (S7 Table). Both knockdowns led to a significant reduction of *VGF* expression in PC3 (one-sided t-tests: $P < 0.0001$). (c) Clonogenic survival analysis for irradiation doses from 0 to 6 Gy (S7 Table). *VGF* knockdowns moderately radiosensitize PC3 cells in comparison to the negative control (e.g. one-sided t-tests for siRNA VGF #2 vs. negative control: $P = 0.04881$ at 2 Gy and $P = 0.02918$ at 4 Gy). Error bars represent the standard error of the mean. Three biological replicates were considered for this analysis. The increase of the orange curve at 6 Gy compared to 4 Gy can be explained by the very low plating efficiency for siRNA VGF #2 knockdowns of *VGF* (d) leading to too few surviving cells for a robust evaluation at 6 Gy. (d) Plating efficiency of PC3 cells before irradiation. Error bars represent the standard error of the mean. Three biological replicates were considered.
