# Supplementary material for: Network-based analysis of prostate cancer cell lines reveals novel marker gene candidates associated with radioresistance and patient relapse
Source: PLoS Comput Biol. 2019 Nov 4;15(11):e1007460. doi: 10.1371/journal.pcbi.1007460 (PMC6855562; doi:10.1371/journal.pcbi.1007460)
Supplement: S10 Fig — (PDF) [file pcbi.1007460.s011.pdf]

S10 Figure: Western blots and RT-qPCR analysis.

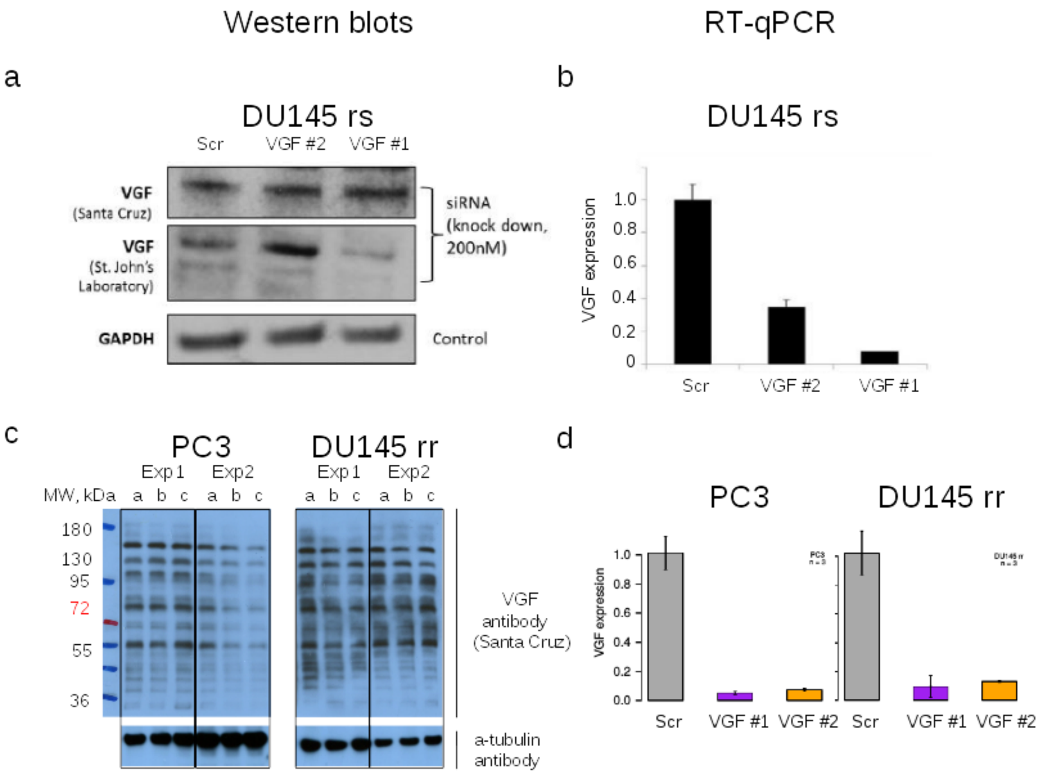

S10 Figure: Analysis of *VGF* expression by Western blots and RT-qPCR analysis. (a) Western blot analysis of VGF siRNA knockdown in DU145 parental radiosensitive cells. Scr: scrambled siRNAs used as negative control, VGF #1 and VGF #2: siRNA knockdowns. Two *VGF* antibodies were tested (anti-VGF Santa Cruz sc-365397, B-8 mouse; St. John's Laboratory, STJ96661, rabbit, polyclonal). GAPDH was used as loading control. Both antibodies gave unspecific bands that were not consistent with the corresponding RT-qPCR data in (b). (b) RT-qPCR analysis of DU145 parental radiosensitive cells transfected with VGF siRNAs for one experiment consisting of three technical replicates. Error bars represent the standard deviation of technical replicates. Efficiency of *VGF* knockdowns by *VGF*-specific siRNAs (VGF #1 and VGF #2) is shown in relation to the *VGF* expression in negative control (Scr: scrambled siRNAs). (c) Western blot analysis of PC3 cells and of DU145 radioresistant cells transfected with *VGF*-specific siRNAs (VGF #1 and VGF #2) considering the *VGF* antibody from Santa Cruz (anti-VGF Santa Cruz sc-365397, B-8 mouse). Cells transfected with scrambled siRNA were used as a control. An anti-alpha-tubulin antibody (Cell Signaling Technology, DM1A, #3873) was used as a loading control. Two independent experiments were performed for each cell line (Exp1 and Exp2), where column 'a' represents the negative control (Scr: scrambled siRNAs), column 'b' represents VGF #1, and column 'c' represents VGF #2 treatment. *VGF* has a tentative molecular weight of 67 kDa indicated by the red line. Unspecific bands were observed in all experiments for both cell lines. Western blot results were not consistent with knockdowns of *VGF* expression confirmed by RT-qPCR analysis (d). (d) RT-qPCR analysis of PC3 cells and of DU145 radioresistant cells transfected with VGF siRNAs. Three biological replicates were performed for each cell line. Efficiency of *VGF* knockdowns by *VGF*-specific siRNAs (VGF #1 and VGF #2) is shown in relation to the *VGF* expression in negative control (Scr: scrambled siRNAs). (a and c) Western blot analysis: Cells were lysed in radioimmunoprecipitation assay (RIPA) buffer (Santa Cruz Biotechnology) and protein concentrations were determined using the bicinchoninic acid (BCA) protein assay kit (Pierce) according to manufacturer's recommendations. All primary antibodies were used at concentrations recommended by the manufacturer followed by incubation with a 1:10,000 dilution of horseradish peroxidase (HRP)-conjugated secondary antibody (Santa Cruz Biotechnology). Used primary antibodies are anti-VGF (St. John's Laboratory, STJ96661, 1:1000), anti-VGF (clone B-8, sc-365397, Santa Cruz Biotechnology, 1:1000), anti-GAPDH (clone FL-335, Santa Cruz Biotechnology, 1:1000) and anti-α-tubulin antibody (DM1A, #387, Cell Signaling Technology). The signal was visualized using the enhanced chemiluminescence detection reagent (GE Healthcare).
