# Supplementary material for: Network-based analysis of prostate cancer cell lines reveals novel marker gene candidates associated with radioresistance and patient relapse
Source: PLoS Comput Biol. 2019 Nov 4;15(11):e1007460. doi: 10.1371/journal.pcbi.1007460 (PMC6855562; doi:10.1371/journal.pcbi.1007460)
Supplement: S11 Fig — (PDF) [file pcbi.1007460.s012.pdf]

S11 Figure: LQ model fits of clonogenic survival.

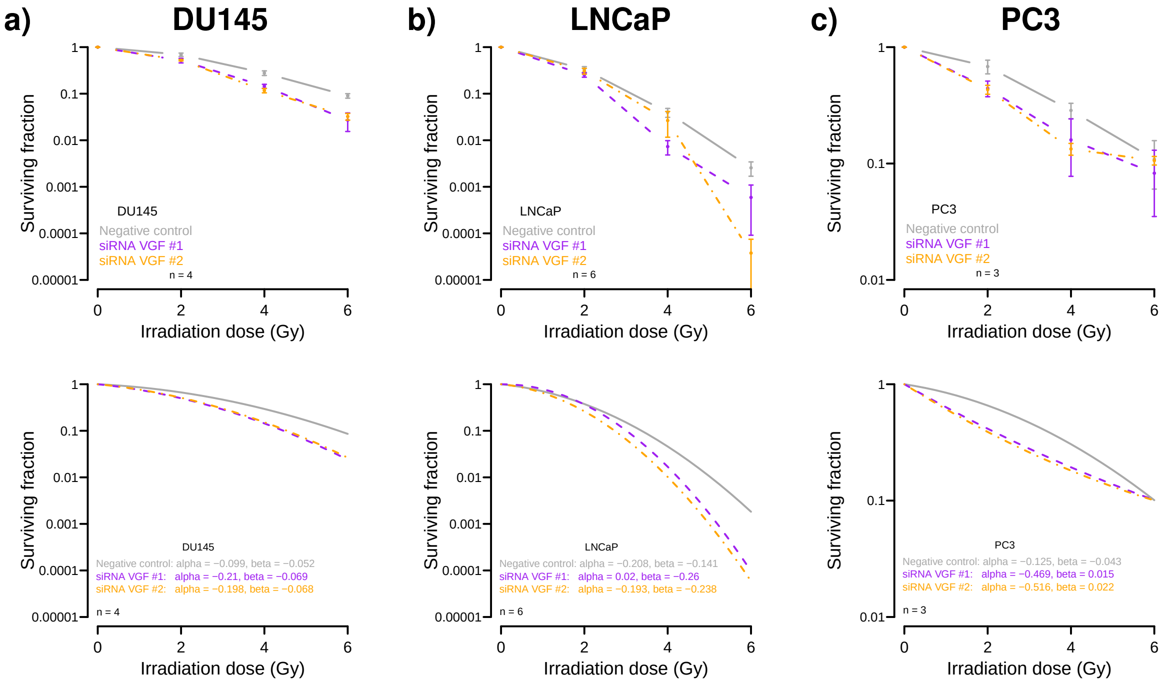

S11 Figure: Comparison of raw clonogenic survival data (first row) to linear-quadratic (LQ) models of clonogenic survival data (second row). LQ models were fitted to the experimentally measured survival fraction of cells of each specific experiment to obtain a model-based clonogenic survival curve that enables to obtain the surviving fraction of cells for a given irradiation dose using the R package CFAssay (Braselmann et al. (2015)). The underlying survival curve is described by $S(d) = exp(\alpha \cdot d + \beta \cdot d^2)$, where d specifies the irradiation dose and α and β represent the model parameters directly learned form the experimental data. Shown are the clonogenic survival curves for the cell lines DU145 (a), LNCaP (b), and PC3 (c) comparing *VGF* knockdowns by siRNA VGF #1 and siRNA VGF #2 to scrambled siRNAs (negative control). The number of biological replicates done for each cell line is denoted by 'n' and the obtained experiment-specific 'alpha' and 'beta' values are shown. Underlying measurements of DU145 and LNCaP were obtained from the parental cell lines (S7 Table).
